# Is liver involvement overestimated in COVID-19 patients? A meta-analysis

**DOI:** 10.7150/ijms.51174

**Published:** 2021-01-18

**Authors:** Gang Li, Yitian Yang, Danyang Gao, Yongxing Xu, Jianwen Gu, Pengfei Liu

**Affiliations:** 1 Department of General Surgery, Peking University Third Hospital, Haidian District, Beijing 100191, China.; 2 Department of Anesthesiology and Perioperative Medicine, Henan Provincial People's Hospital, People's Hospital of Zhengzhou University, Zhengzhou, 450003, China.; 3 Department of Anesthesiology, Capital Medical University affiliated Beijing Shijitan Hospital, Beijing 100038, China.; 4 Department of Nephrology, PLA Strategic Support Force Characteristic Medical Center, Beijing 100101, China.; 5 The Leading Group on COVID-19 Prevention and Control, People's Liberation Army Strategic Support Force Characteristic Medical Center, Beijing 100101, China.; 6 Department of Anesthesiology, Capital Medical University affiliated Beijing Shijitan Hospital, Beijing 100038, China.

**Keywords:** COVID-19, hypertransaminemia, liver injury, alanine aminotransferase, aspartate aminotransferase, meta-analysis

## Abstract

**Background:** Considering transaminase more than the upper limit of normal value as liver injury might overestimate the prevalence of liver involvement in COVID-19 patients. No meta-analysis has explored the impact of varied definitions of liver injury on the reported prevalence of liver injury. Moreover, few studies reported the extent of hypertransaminasemia stratified by COVID-19 disease severity.

**Methods:** A literature search was conducted using PubMed and Embase. The pooled prevalence of liver injury and hypertransaminasemia was estimated.

**Results:** In total, 60 studies were included. The overall prevalence of liver injury was 25%. Compared to subgroups with the non-strict definition of liver injury (33%) and subgroups without giving detailed definition (26%), the subgroup with a strict definition had a much lower prevalence of liver injury (9%). The overall prevalence of alanine aminotransferase (ALT) and aspartate aminotransferase (AST) elevation was 19% and 22%. The prevalence of elevated ALT and AST were significantly higher in severe COVID-19 cases compare to non-severe cases (31% vs 16% and 44% vs 11%). In critically ill and fatal cases, no difference was found in the prevalence of elevated ALT (24% vs 30%) or AST (54% vs 49%). Sensitivity analyses indicated that the adjusted prevalence of ALT elevation, AST elevation, and liver injury decreased to 14%, 7%, and 12%.

**Conclusion:** The overall prevalence of liver injury and hypertransaminasemia in COVID-19 patients might be overestimated. Only a small fraction of COVID-19 patients have clinically significant liver injury. The prevalence of hypertransaminasemia was significantly higher in severe COVID-19 cases compare to non-severe cases. Hence, in severe COVID-19 patients, more attention should be paid to liver function tests.

## Introduction

The novel severe acute respiratory syndrome coronavirus 2 (SARS-CoV-2) virus, which causes coronavirus disease 2019 (COVID-19), was discovered in China in December 2019 and quickly spread into an ongoing global pandemic 1, 2. COVID-19 has continued to spread for the greater part of a year and the second wave of COVID-19 has been under way around the world 3.

A growing body of research has reported that some patients with COVID-19 have abnormal liver function tests and liver injury 4-6. There are already several systematic reviews and meta-analyses addressing this topic 7-9. However, few of these studies have reported the pooled prevalence of transaminase elevation and liver injury, and when reported, the results have been inconsistent 10, 11.

The clinical manifestation of COVID-19 ranges from mild to critically ill and fatal cases. Patients with severe COVID-19 have a higher risk of liver involvement 11. To date, there is a gap in knowledge regarding the extent of liver injury and increased transaminase levels stratified by COVID-19 disease severity. Moreover, we think it is not rigorous that some investigators considered the transaminase more than the upper limit of normal value as liver injury 11. In addition, because elevated transaminase might be due to myocardial injury or muscle injury induced by SARS-CoV-2 infection 12, 13, the prevalence of liver injury might be overestimated. There is a lack of a uniform definition of acute liver injury caused by COVID-19. One previous study defined liver injury in COVID-19 as an ALT or AST level greater than 3 times the upper limit of normal 14. By contrast, one study indicated that liver injury was diagnosed according to the elevation of bilirubin and aminotransferase 15. The definitions of liver injury have a major impact on the results of pooled analysis, however, no meta-analysis explored this.

Therefore, we conducted this meta-analysis of clinical studies on the COVID-19 to comprehensively explore the prevalence of transaminase elevation and liver injury in COVID-19 patients. We analyzed the data based on the severity of the COVID-19 to further discuss the liver involvement in different populations. More importantly, based on the different definitions of liver injury, we conducted subgroup analysis to explored the impact of varied definitions of liver injury on the prevalence of liver injury.

## Methods

This meta-analysis followed the preferred reporting items for systematic reviews and meta-analyses (PRISMA) statement (Supplementary Table 1) 16 and was registered with the International Prospective Register of Systematic Reviews (PROSPERO) and assigned registration number CRD42019120201. Moreover, the protocol of this meta-analysis has been published 9.

### Search strategy and selection criteria

We searched PubMed and Embase electronic databases on April 13, 2020, for potentially relevant studies from December 2019 using relevant words and medical subject headings as follows: severe acute respiratory syndrome coronavirus 2, SARS-CoV-2, sars cov 2, 2019-nCoV, 2019 novel CoV, 2019 novel coronavirus, coronavirus disease 19, coronavirus disease 2019, COVID-19, COVID-2019, and novel coronavirus-infected pneumonia (Supplementary Table 2). The reference list of the eligible articles and relevant reviews were manually searched to identify additional studies. There were no language restrictions.

Two independent reviewers (PL and GL) assessed the eligibility of studies with a standardized approach. Discrepancies were resolved by discussion with a third individual (YX). Studies in languages other than English or Chinese were translated into English using Google Translate. When there were multiple studies from the same cohort, the study with the largest sample size was used. If the sample size did not vary, the most recent paper was used.

Two authors (PL and GL) independently extracted data into a standardised excel spreadsheet, including study characteristics, prevalence and definition of acute liver injury, and prevalence of elevated alanine aminotransferase (ALT) and aspartate aminotransferase (AST) in patients with COVID-19. Data on the impact of acute liver injury on the clinical course of COVID-19 disease and prognosis were collected if available. Published observational studies reporting the prevalence of acute liver injury and/or hypertransaminasemia were included. Single case studies and studies with less than 10 participants were excluded. Although we were not interested in interventions, we included nonrandomized analyses performed in populations derived from randomised controlled trials.

### Assessing the risk of bias

The risk of bias of the included studies was assessed independently by two reviewers (PL and GL) using the Joanna Briggs Institute's critical appraisal checklist 17. Any disagreement was discussed by consultation or resolved by a third investigator (YX).

### Outcomes and definitions

The prevalence of acute liver injury in patients with COVID-19 was the main outcome. The prevalence of elevated ALT and AST in patients with COVID-19 were secondary outcomes. Acute liver injury and elevated ALT and AST were defined based on criteria used in the individual studies. Based on previous studies,^14^, ^18^-^20^ we adopted a relatively strict definition of acute liver injury as ALT and/or AST higher than 3-fold of the upper limit of normal (ULN), or alkaline phosphatase and/or total bilirubin (TBIL) higher than 2-fold of the ULN. In studies that considered any ALT, AST, alkaline phosphatase, and/or TBIL level higher than the ULN as liver injury, the definition was considered not strict or insufficiently rigorous. As in a previous study,^11^ disease severity of COVID-19 was defined based on criteria used in the individual studies; meanwhile, COVID-19 patients requiring admission to the intensive care unit or with acute respiratory distress syndrome were classified as severe cases 11. Fatal cases were also considered as severe cases.

### Statistical analysis

The pooled prevalence of hypertransaminasemia and acute liver injury was estimated using a random-effects model 21. Forest plots were generated to display prevalence with corresponding 95% confidence intervals (CIs). The heterogeneity between studies was investigated statistically using the chi-square test and I^2^ statistic. I^2^ values of 25%, 50%, and 75% corresponded to low, medium, and high levels of heterogeneity, respectively 22. Subgroup analyses were undertaken based on the severity of COVID-19 and age (adult vs children). The impact of the various definitions of liver injury on the reported prevalence of liver injury was also explored. Sensitivity analyses were performed using the Duval and Tweedie nonparametric “trim and fill” procedure 23, 24. Funnel plots, Egger's regression asymmetry test, and Begg's test were used to evaluate publication bias 25, 26. A two-sided *p*-value < 0.05 was considered significant for all analyses. All analyses were performed using Stata software v 15.0 (StataCorp, College Station, TX, USA).

## Results

**Figure 1:**
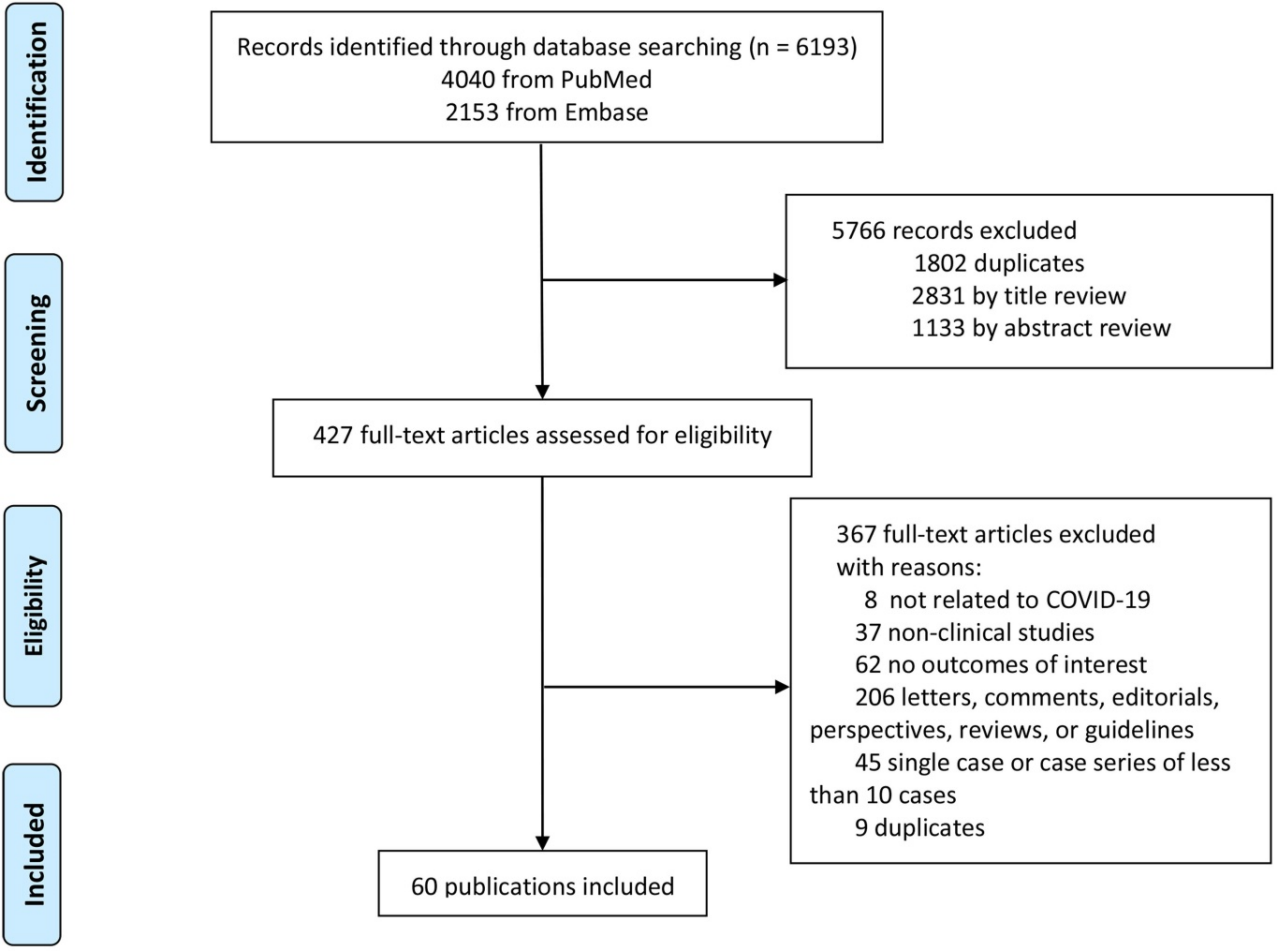
Study selection.

Initially, 6193 publications were identified, 1802 of which were duplicates and excluded. Subsequently, 4328 articles were excluded after browsing the titles, abstracts, and full-text reviews for relevancy. Finally, a total of 60 1, 4-6, 12, 14, 15, 27-79 articles that met the inclusion criteria were included in the study (Fig. 1).

The main characteristics of patients and studies included in the meta-analysis were shown in Supplementary Table 3. The risk of bias of the included studies was shown in Supplementary Table 4.

**Figure 2:**
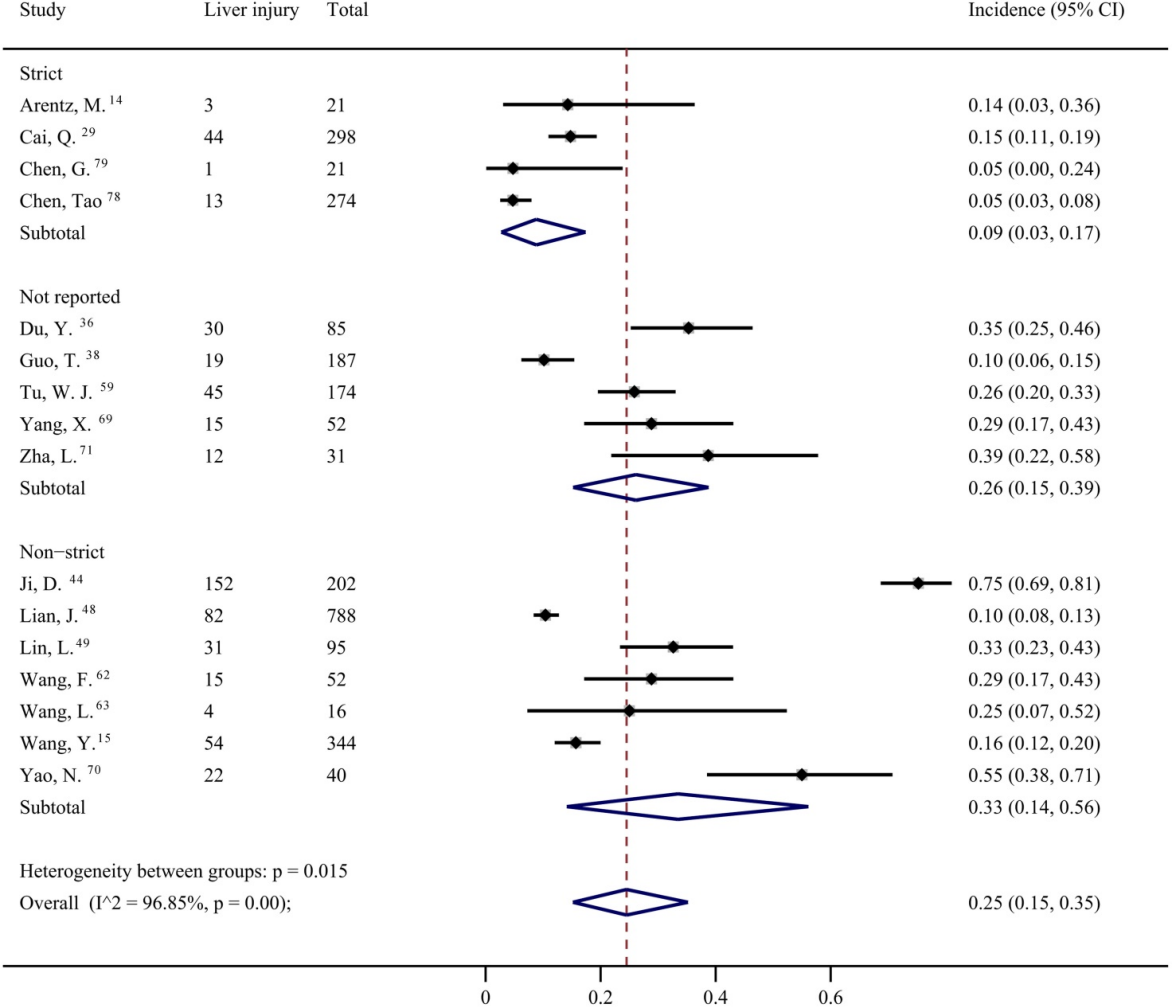
Estimated incidence prevalence of liver injury based on definition.

From 16 studies 14, 15, 29, 36, 38, 44, 48, 49, 59, 62, 63, 69-71, 78, 79, the overall prevalence of liver injury in the COVID-19 patients was 25% (95% CI: 0.15, 0.35) with significant heterogeneity among the studies (*I^2^*=96.85%), as shown in Figure 2 and Supplementary Table 5. The definition of liver injury varied among the studies. Four studies were considered to have strict definitions 14, 29, 78, 79, seven were considered to have non-strict definitions 15, 44, 48, 49, 62, 63, 70, and five did not provide definitions 36, 38, 59, 69, 71. Subgroup analysis showed a significant difference in the prevalence of liver injury among the three subgroups according to different type of definition (*p* = 0.015; Figure 2). The prevalence of liver injury in subgroups with strict, non-strict, and unreported definitions were 9% (95% CI: 0.03, 0.17), 33% (95% CI: 0.14, 0.56), and 26% (95% CI: 0.15, 0.39), respectively (Figure 2). In subgroup with strict definition, the prevalence of liver injury was 17% (95% CI: 0.04, 0.34) in patients with severe COVID-19, with significant heterogeneity among the studies (*I^2^* = 83.36%; Supplementary Figure 1).

**Figure 3:**
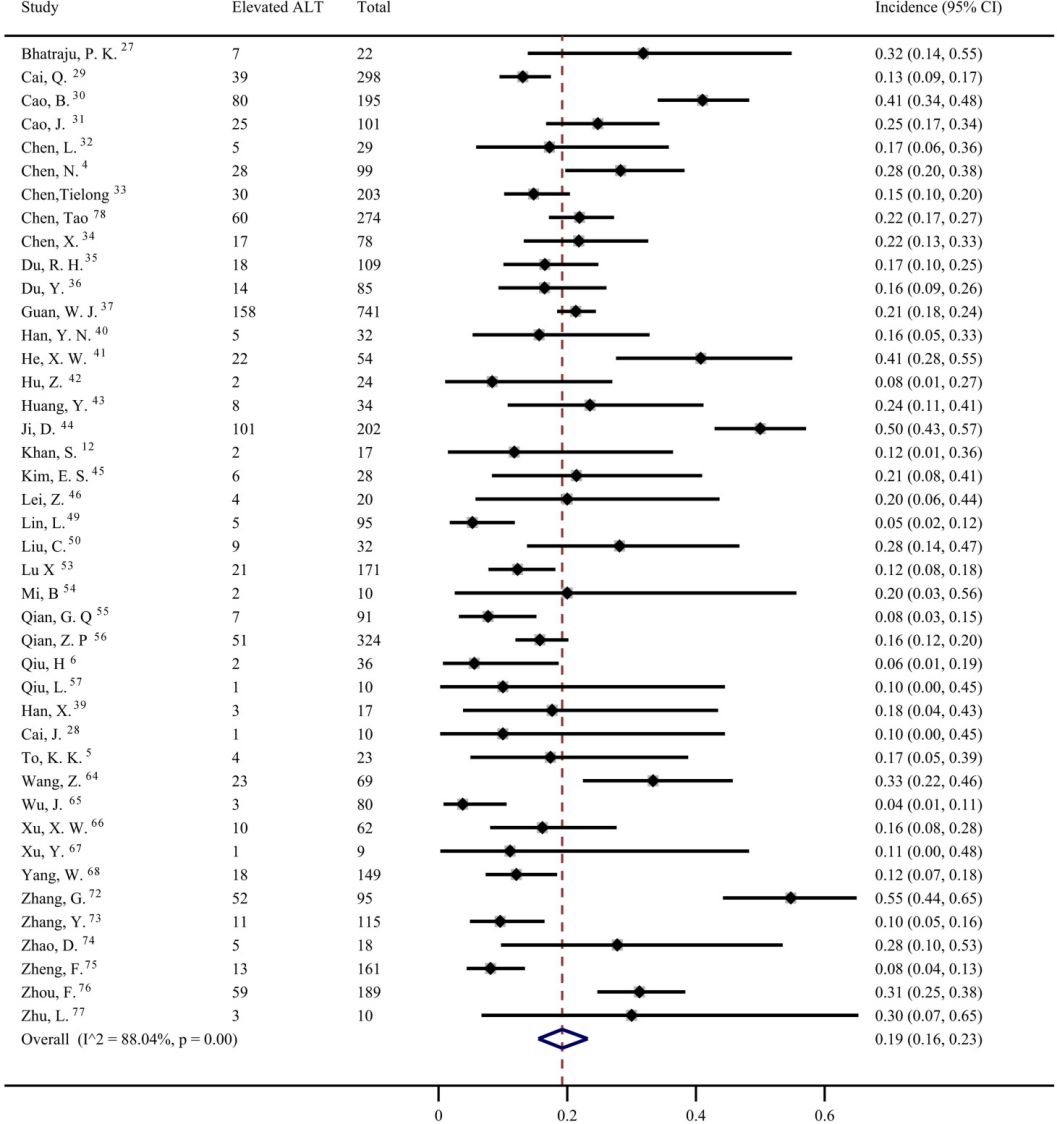
The pooled estimate of elevated alanine aminotransferase in patients with COVID-19.

**Figure 4:**
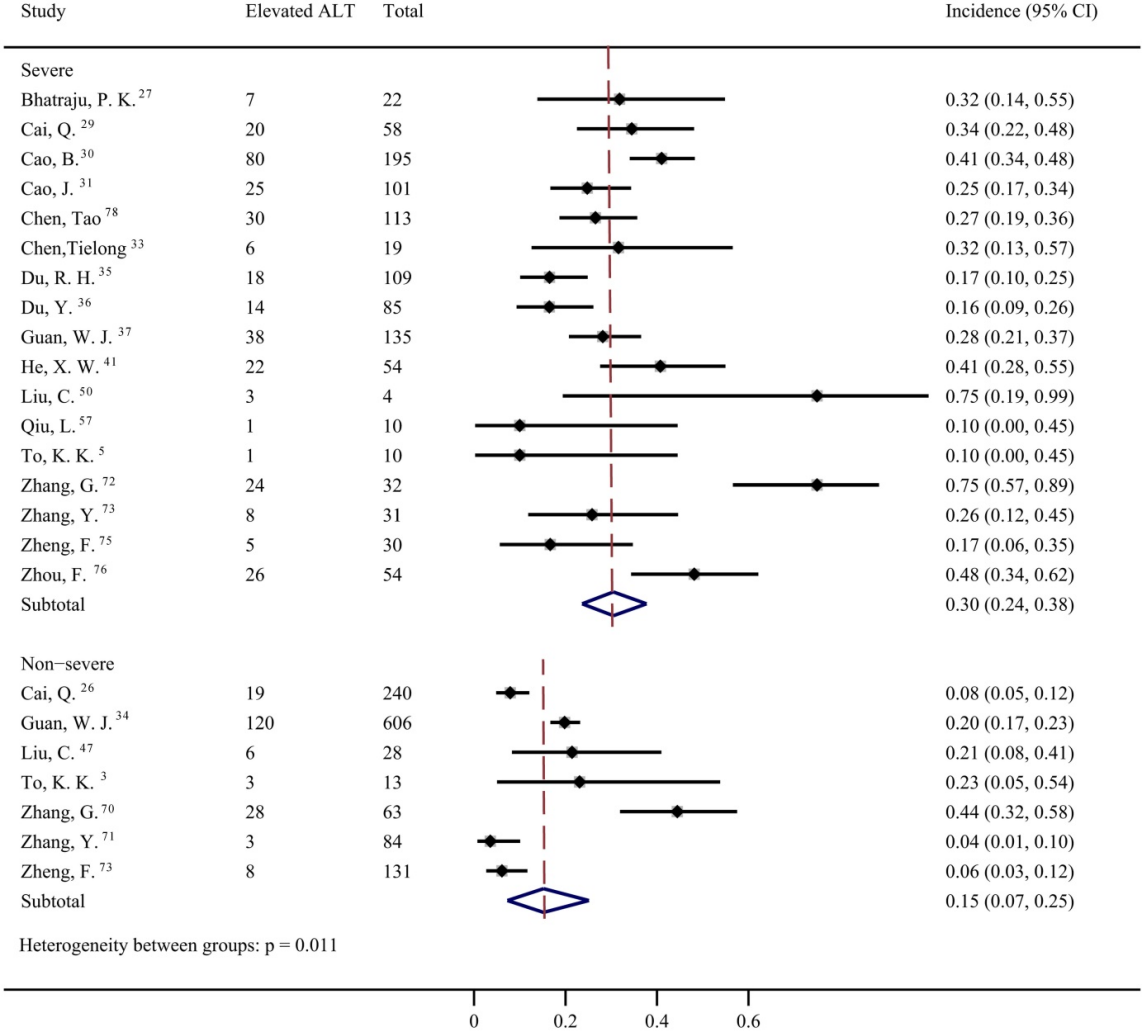
Pooled estimate of elevated alanine aminotransferase in severe and non-severe patients with COVID-19.

Data from 42 studies 4-6, 12, 27-37, 39-46, 49, 50, 53-57, 64-68, 72-78 were pooled for meta-analysis of elevated ALT levels in COVID-19 patients, resulting in prevalence of 19% (95% CI: 0.16, 0.23; Figure 3). Significant heterogeneity among the studies was detected (*I^2^*=88.04%; *p* < 0.001). Subgroup analyses revealed that the prevalence of elevated ALT in patients with severe COVID-19 was 30% (95% CI: 0.24, 0.38), which was significantly higher than that in patients with non-severe cases (15%; 95% CI: 0.07, 0.25) (*p* = 0.011 for subgroup difference; Figure 4). However, there was no significant difference in the prevalence of elevated ALT levels between critically ill patients (24%; 95% CI: 0.10, 0.41) and fatal cases (30%; 95% CI: 0.20, 0.41) (*p* = 0.611 for subgroup difference; Supplementary Figure 2).

**Figure 5:**
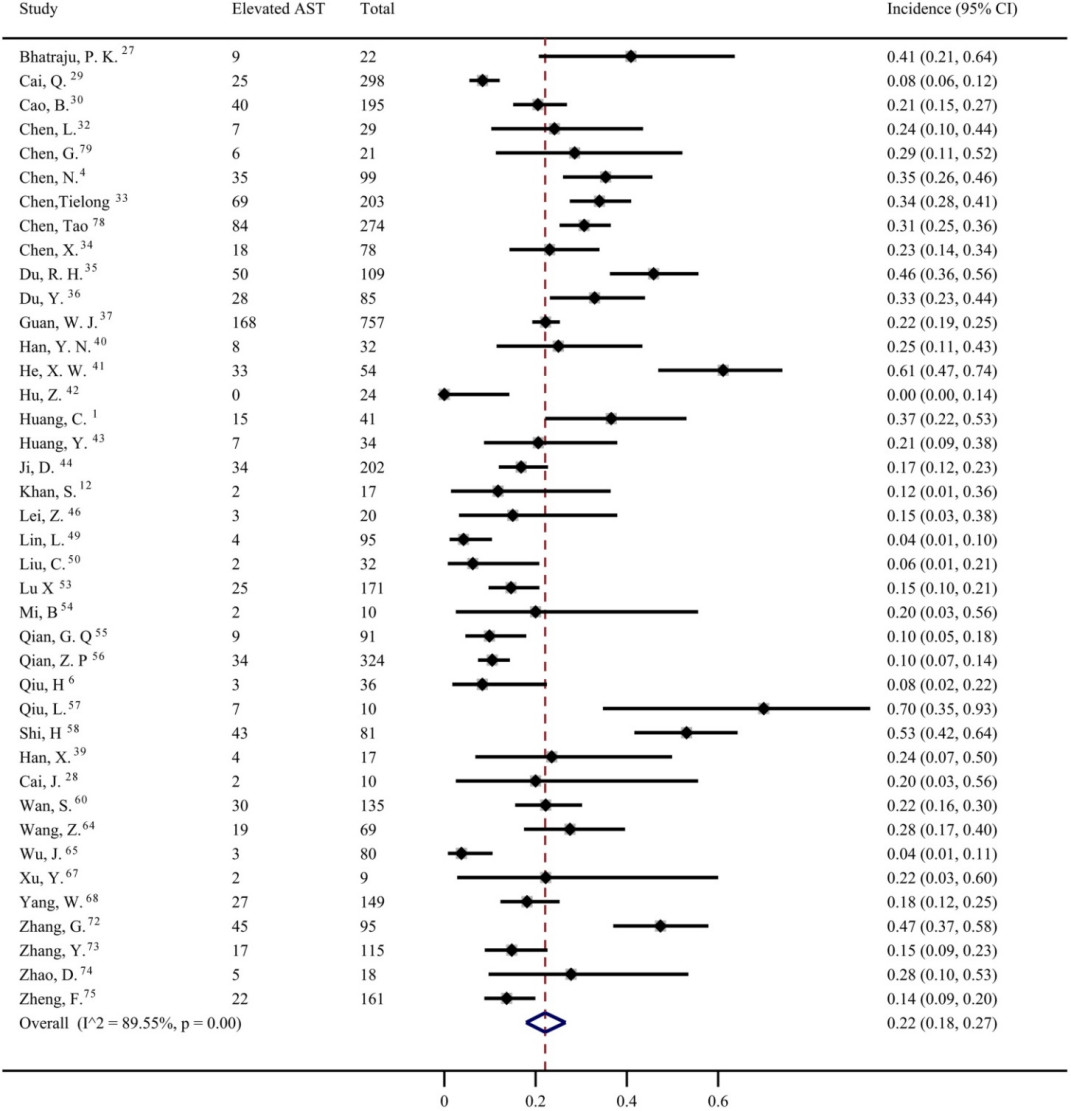
The pooled estimate of elevated aspartate aminotransferase in patients with COVID-19.

**Figure 6:**
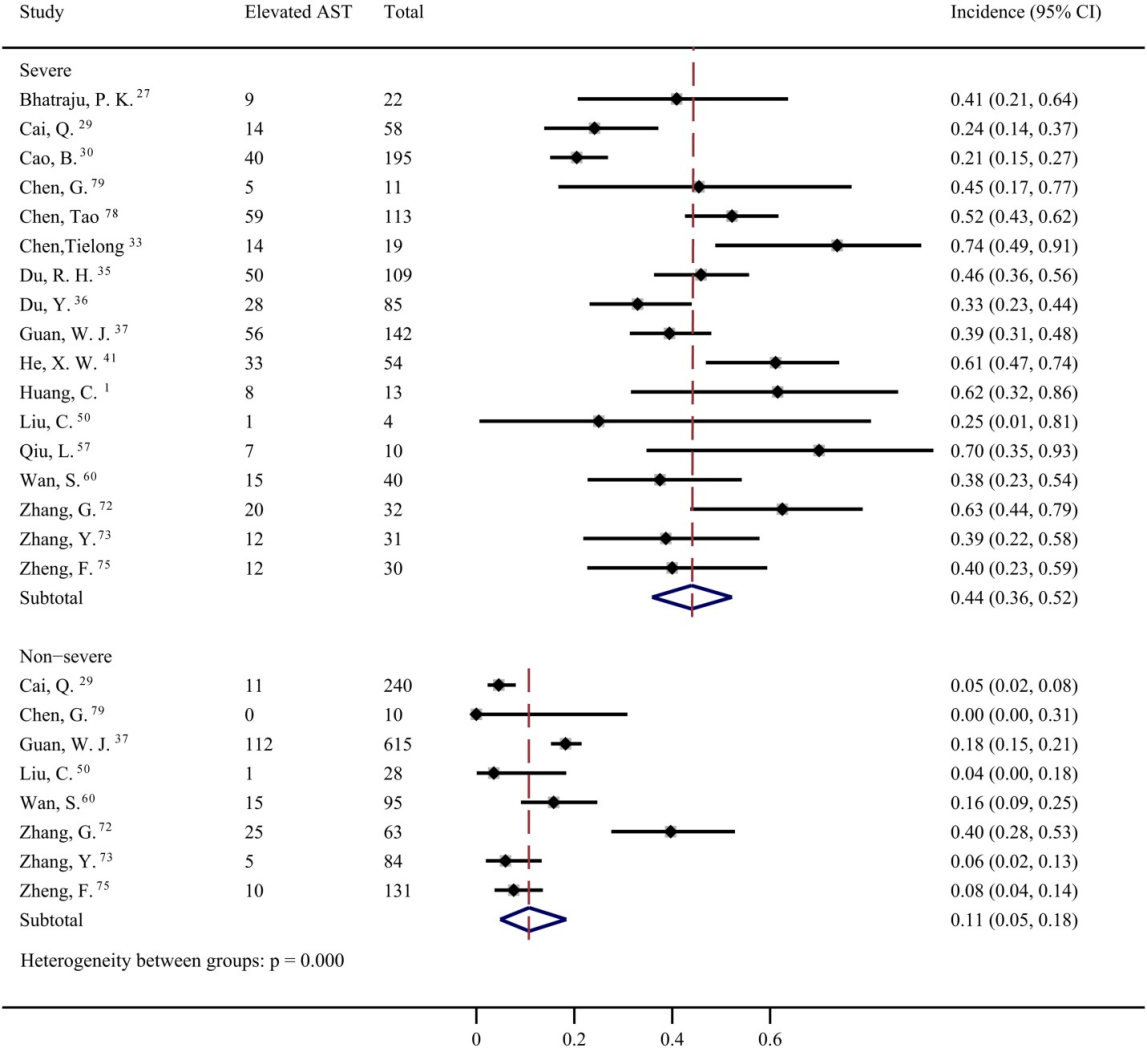
Pooled estimate of elevated aspartate aminotransferase in severe and non-severe patients with COVID-19.

Forty studies 1, 4, 6, 12, 27-30, 32-37, 39-44, 46, 49, 50, 53-58, 60, 64, 65, 67, 68, 72-75, 78, 79 were included to evaluate the prevalence of elevated AST in COVID-19 patients. The pooled prevalence was 22% (95% CI: 0.18, 0.27; Figure 5) with significant heterogeneity noted among the studies (*I^2^*=89.55%; *p* < 0.001). Subgroup analyses showed that the patients with severe COVID-19 had a significantly higher risk of elevated AST than non-severe COVID-19 patients (44%; 95% CI: 0.36, 0.5 vs 11%; 95% CI: 0.05, 0.18) (*p* < 0.001 for subgroup difference; Figure 6). However, there was no significant difference in the prevalence of elevated AST between the critically ill patients (54%; 95% CI: 0.37, 0.72) and the fatal cases (49%; 95% CI: 0.36, 0.61) (*p* =0.601 for subgroup difference; Supplementary Figure 3).

Four studies 47, 51, 52, 61 reported the prevalence of elevated transaminase (a mixture of data on elevated ALT or AST), but neither exact number of elevated ALT nor AST was available in these studies. Hence, we presented a narrative synthesis. These four studies came from China and included relatively fewer subjects (ranging from 10 to 31. Only two studies 51, 52 reported the normal range of ALT and AST. The prevalence of elevated transaminase ranged from 20% to 48%.

The overall prevalence of elevated ALT in paediatric patients was significantly lower than that in adult patients (10% [95% CI: 6-14] vs 21% [95% CI 13-30]; *p* = 0.032 for subgroup difference; Supplementary Figure 4). The prevalence of elevated AST in paediatric patients was also lower than that in adult patients, however, the difference did not reach significance (14% [95% CI: 7-22] vs 24% [95% CI: 16-33]; *p* = 0.094 for subgroup difference; Supplementary Figure 5). We further compared the prevalence of elevated ALT and AST in pediatric patients to severe adult and non-severe adult COVID-19 patients by subgroup analysis (Supplementary Figure 6 and 7). The prevalence of elevated ALT in paediatric patients and adults with severe and non-severe COVID-19 were 10% (95% CI: 6, 14%), 27% (95% CI: 16, 39%), and 6% (95% CI: 3, 12%), respectively and the prevalence of elevated AST were 14% (95% CI: 7, 22%), 39% (95% CI: 20, 59%), and 8% (95% CI: 4, 14%), respectively. All of the differences in elevated ALT and AST among the subgroups were significant (*p* < 0.001 and *p* = 0.001 for subgroup difference, respectively). We found that no study reported the prevalence of liver injury in children with COVID-19.

Although the funnel plot of ALT was slightly asymmetric (Supplementary Figure 8), neither the Begg's nor Egger's tests were statistically significant (*p* = 0.111 and 0.080, respectively). The funnel plot of AST displayed a clear asymmetry (Supplementary Figure 9). The Begg's test of AST results showed no statistical significance (*p* = 0.907), but the Egger's test showed statistical significance (*p* < 0.001). Through visual inspection, the funnel plots of liver injury showed obvious asymmetry (Supplementary Figure 10). The Egger's test showed statistical significance (*p* = 0.045) but the Begg's test was not statistically significant (*p* = 0.126).

Subsequently, we performed sensitivity analyses by the Duval and Tweedie nonparametric “trim and fill” procedure. As a result of the ALT sensitivity analysis, 13 studies were deemed as missing and were imputed into the trim and fill plot (Supplementary Figure 11). The adjusted point estimate decreased to 14% (95% CI: 10%-17%) under the random effects model. For AST, 20 studies were missing, which were entered using the funnel plot (Supplementary Figure 12). After adjusting, the point estimate was reduced to 7% (95% CI: 3%-12%) under the random effects model. For liver injury, after adding 7 studies into the plot, the adjusted point estimate was reduced to 12% (95% CI: 3%-21%) under the random effects model (Supplementary Figure 13).

One study conducted by Yao 70 indicated that there were more severe cases in COVID-19 patients with liver injury compared to those without liver injury (77.3% vs 27.8%). Among the studies included in the present meta-analysis, three discussed the association between elevated transaminase and the prognosis of patients. Chen 33 reported that the patients with elevated AST (AST > 40 U/L) tended to have a higher risk of death but without a significant difference (odds ratio: 2.04; 95% CI: 0.56, 7.38; *p* = 0.28). Zhou 76, in a retrospective study, found that the odds of death were higher in patients with elevated ALT (ALT>40 U/L) according to univariable analysis (odds ratio: 2.87; 95% CI: 1.48, 5.57; *p* <0.01), while elevated ALT (ALT > 40 U/L) was not associated with an increased risk of death by further multivariate regression analysis (*p* > 0.05). However, Zhang et al. 72 found that both higher ALT and AST activity were related to the composite endpoint (including mechanical ventilation, admission to the intensive care unit, and death) (*p* < 0.05).

## Discussion

In this comprehensive meta-analysis, the overall prevalence of liver injury defined by the criteria used in the individual studies was 25%. By subgroup analysis, we found the various definitions of liver injury had a significant impact on the reported prevalence of liver injury. The prevalence of clinically significant liver injury defined by strict criteria was only 9%. Sensitivity analyses indicated that the overall prevalence of liver injury and increased aminotransferase might be overestimated.

The underlying mechanisms of COVID-19-related liver injury remain unclear. Clinically, liver injury might be associated with pre-existing liver diseases, viral infection, drug toxicity, and systemic inflammation 80-84. Pneumonia-associated hypoxia might also contribute to liver injury 84. Some studies report that the angiotensin-converting enzyme 2 (ACE 2), the receptor of SARS-CoV-2, mainly located in the lungs, is also widely expressed in liver cells 80. Besides, pulmonary hypertension and reduced right heart function can be observed in COVID-19 patients 85-87. Acute heart failure was one of the most common critical complications during the exacerbation of COVID-19 78. Liver has a high metabolic activity and perfusion rate. Liver injury might be brought about by acute circulatory changes, in the setting of which the liver's compensatory mechanism is being insufficient 88. Hence, liver injury in COVID-19 patients might also be related to haemodynamic issues.

Currently, there is no uniform definition of liver injury for COVID-19 patients. Several studies considered the abnormal liver function tests in the COVID-19 patients as the liver injury 11, 15, but this is not a sufficient criterion because mild aminotransferase elevation could also occur in COVID-19 patients with myocardial damage and/or muscle injury 12, 13. By contrast, one study defined liver injury in COVID-19 as an ALT or AST level greater than 3 times the ULN 14. Hence, based on previous studies and definitions of other types of liver injury (such as drug-induced liver injury, liver injury in HIV, and liver injury in adult dengue infection) 14, 18-20, we adopted a relatively strict definition of acute liver injury, which was ALT and/or AST higher than 3-fold of the ULN, or alkaline phosphatase and/or TBIL higher than 2-fold of the ULN 9.

The overall prevalence of liver injury based on the definition included in the individual studies was 26%, higher than the 19% reported by Mao et al.^11^ Besides, our results suggested that the definition of liver injury has a major impact on the reported prevalence in patients with COVID-19. According to the stricter definition, we found the prevalence of liver injury was only 9%.

Although previous meta-analyses have addressed abnormal liver function 7, 8, 10, 11, 74, 89-92, few studies have investigated the prevalence of elevated aminotransferase 10, 11. Several previous meta-analyses 7, 8, 89, estimated weighted mean difference or standardised mean difference of liver function measure in COVID-19 patients, but not reported the prevalence of elevated aminotransferase. Mao et al. indicated that the pooled prevalence of elevated ALT was 18% from 14 studies and elevated AST was 21% by combining 14 studies 11. Another study reported that the pooled prevalence estimates of elevated ALT and elevated AST were both 15.0% 10. In this meta-analysis, 17 and 16 studies were included when performing the pooled prevalence of ALT and AST, respectively 10. In the present meta-analysis, the overall prevalence of elevated ALT and AST was 19% and 22%, respectively. These results are similar to those in the study conducted by Mao et al. 11. However, the present study included more studies. Specifically, there were a total of 42 studies that reported the prevalence of elevated ALT and 40 studies that reported the prevalence of elevated AST. However, abnormal ALT and AST level in COVID-19 patients need to be interpreted cautiously 93, 94. As we stated, not all abnormal liver function tests mean that patients with COVID-19 have liver injury, and elevated aminotransferases might partly result from myocardial injury and muscle injury 94.

There is limited data from meta-analyses on the prevalence of elevated transaminase levels based on COVID-19 severity. In Mao et al., odds ratios were used to describe the probability of abnormal liver chemistry, including increased ALT and AST levels according to disease severity, and found a higher risk of abnormal liver chemistry in patients with severe COVID-19 11. In the present study, we provided the exact magnitude of the prevalence of elevated ALT and the prevalence of elevated AST in severe and non-severe COVID-19 patients, which were 30% versus 15%, and 44% versus 11%, respectively. We also provided the prevalence of elevated ALT and the prevalence of elevated AST in critically ill cases as well as fatal cases, and found the differences were not significant between the two subgroups. Because the overall prevalence of elevated ALT and AST is influenced by the proportion of studies enrolling patients with severe COVID-19 in meta-analyses, we think that epidemiologic data are more valuable when reported for different subpopulations stratified by severity of COVID-19.

The prevalence of elevated transaminase levels in paediatric patients with COVID-19 showed that elevated ALT in this subpopulation were significantly lower than those of adult patients, which is consistent with a previous study 11. However, unlike the study conducted by Mao et al. 11, the difference in AST was not significant. We further compared the prevalence of elevated ALT and the prevalence of elevated AST in paediatric patients compared to adults with severe and non-severe COVID-19. The differences seemed to be driven by adult patients with severe COVID-19. Therefore, we suppose that no significant difference in the elevated transaminase exists between paediatric and adults with non-severe COVID-19, because most paediatric patients have clinically milder symptoms and favourable prognosis 95, 96. However, due to inadequate studies with adult patients with non-severe COVID-19, the corollary needs to be further confirmed.

Among the studies included in the present meta-analysis, three discussed the association between elevated transaminase and the prognosis of patients, and the results were inconsistent partially because of different analysis methods and outcome indicators 33, 72, 76. Recently, one study evaluated the association of mild vs severe liver injury with mortality in COVID-19 patients and found that severe liver injury in this cohort was associated with multiple-organ failure and mortality. Whereas mild liver injury was not related to poor outcomes 97. Hence, although our meta-analysis showed that the prevalence of liver injury defined by strict criteria was only 9%, severe liver injury is worthy of note. More research is needed to address whether transaminase elevation or liver injury is an independent risk factor for death in COVID-19 patients.

Sensitivity analyses were performed to verify the robustness of the results regarding the overall prevalence of increased aminotransferase and liver injury. As a result, the adjusted point estimates were remarkably different from the unadjusted ones. The adjusted prevalence of increased ALT, increased AST, and liver injury in the COVID-19 patients were all decreased, which indicates that the overall prevalence of hypertransaminemia and liver injury in patients with COVID-19 might be overestimated.

Compared to previous studies, there are several strengths in our study. First, we show evidence that the definition of liver injury might have a significant impact on the reported prevalence of liver injury in COVID-19 patients. Second, we found that the liver injury defined with strict criteria might be not common. Third, a sensitivity analysis was performed to estimate the robustness of the results obtained by the trim and fill method, which showed that both hypertransaminemia and liver injury may be overestimated in COVID-19 patients.

This meta-analysis also had some limitations. First, as the research on COVID-19 progresses rapidly, the most recently published literature could not be included. Second, there was large heterogeneity among the studies where the normal range of aminotransferases and the definition of liver injury were not consistent. Third, significant publication bias in the meta-analysis was found. Finally, most studies had a small sample size.

In conclusion, our study found the definition of liver injury might have a significant impact on the reported prevalence in COVID-19 patients and provided the exact magnitude of prevalence of elevated ALT and AST in non-severe and severe COVID-19 patients, as well as critically ill and fatal cases. Our study showed that only a small fraction of COVID-19 patients had clinically significant liver injury defined by strict criteria. Sensitivity analyses indicated that the overall prevalence of hypertransaminemia and liver injury might be overestimated. However, in patients with severe COVID-19, more attention should be paid to liver function tests.

## Supplementary Material

Supplementary figures and tables.

## Author Contributions

Yongxing Xu, Jianwen Gu, and Pengfei Liu conceived the study. Jianwen Gu supervised the whole study and manuscript editing. Danyang Gao, Yitian Yang, and Gang Li drafted the manuscript. Gang Li, Pengfei Liu, and Yongxing Xu extracted the data. Yongxing Xu, Gang Li, Pengfei Liu and Yitian Yang analysed the data. Yitian Yang, Gang Li, Danyang Gao, Yongxing Xu, and Pengfei Liu contributed to the materials/analysis tools. All authors read and approved the final manuscript.
