# Supplementary material for: Is liver involvement overestimated in COVID-19 patients? A meta-analysis
Source: Int J Med Sci. 2021 Jan 18;18(5):1285–96. doi: 10.7150/ijms.51174 (PMC7847626; doi:10.7150/ijms.51174)

Supplementary Table 1. PRISMA-P (Preferred Reporting Items for Systematic review and Meta-Analysis Protocols) 2015 checklist: recommended items to address in a systematic review protocol

Section/topic	#	Checklist item	Reported on page #
**TITLE**			
Title	1	Identify the report as a systematic review, meta-analysis, or both.	1
**ABSTRACT**			
Structured summary	3	Provide a structured summary including, as applicable: background; objectives; data sources; study eligibility criteria, participants, and interventions; study appraisal and synthesis methods; results; limitations; conclusions and implications of key findings; systematic review registration number.	3
**INTRODUCTION**			
Rationale	5	Describe the rationale for the review in the context of what is already known.	4
Objectives	6	Provide an explicit statement of questions being addressed with reference to participants, interventions, comparisons, outcomes, and study design (PICOS).	4
**METHODS**			
Protocol and registration	6	Indicate if a review protocol exists, if and where it can be accessed (e.g., Web address), and, if available, provide registration information including registration number.	5
Eligibility criteria	6	Specify study characteristics (e.g., PICOS, length of follow-up) and report characteristics (e.g., years considered, language, publication status) used as criteria for eligibility, giving rationale.	5
Information sources	7	Describe all information sources (e.g., databases with dates of coverage, contact with study authors to identify additional studies) in the search and date last searched.	5

Search	6	Present full electronic search strategy for at least one database, including any limits used, such that it could be repeated.	5
Study selection	7	State the process for selecting studies (i.e., screening, eligibility, included in systematic review, and, if applicable, included in the meta-analysis).	5
Data collection process	7	Describe method of data extraction from reports (e.g., piloted forms, independently, in duplicate) and any processes for obtaining and confirming data from investigators.	5
Data items	7	List and define all variables for which data were sought (e.g., PICOS, funding sources) and any assumptions and simplifications made.	5,6
Risk of bias in individual studies	8	Describe methods used for assessing risk of bias of individual studies (including specification of whether this was done at the study or outcome level), and how this information is to be used in any data synthesis.	6
Summary measures	8	State the principal summary measures (e.g., risk ratio, difference in means).	6
Synthesis of results	8	Describe the methods of handling data and combining results of studies, if done, including measures of consistency (e.g., I^2) for each meta-analysis.	7
Risk of bias across studies	8	Specify any assessment of risk of bias that may affect the cumulative evidence (e.g., publication bias, selective reporting within studies).	7
Additional analyses	9	Describe methods of additional analyses (e.g., sensitivity or subgroup analyses, meta-regression), if done, indicating which were pre-specified.	7
**RESULTS**			
Study selection	9	Give numbers of studies screened, assessed for eligibility, and included in the review, with reasons for exclusions at each stage, ideally with a flow diagram.	7
Study characteristics	10	For each study, present characteristics for which data were extracted (e.g., study size, PICOS, follow-up period) and provide the citations.	7

Risk of bias within studies	9-14	Present data on risk of bias of each study and, if available, any outcome level assessment (see item 12).	7
Results of individual studies	9-14	For all outcomes considered (benefits or harms), present, for each study: (a) simple summary data for each intervention group (b) effect estimates and confidence intervals, ideally with a forest plot.	7-9
Synthesis of results	9-14	Present results of each meta-analysis done, including confidence intervals and measures of consistency.	7-9
Risk of bias across studies	9-14	Present results of any assessment of risk of bias across studies (see Item 15).	9
Additional analysis	9-14	Give results of additional analyses, if done (e.g., sensitivity or subgroup analyses, meta-regression [see Item 16]).	9-10
**DISCUSSION**			
Summary of evidence	14-19	Summarize the main findings including the strength of evidence for each main outcome; consider their relevance to key groups (e.g., healthcare providers, users, and policy makers).	10-14
Limitations	19	Discuss limitations at study and outcome level (e.g., risk of bias), and at review-level (e.g., incomplete retrieval of identified research, reporting bias).	14
Conclusions	19-20	Provide a general interpretation of the results in the context of other evidence, and implications for future research.	14
**FUNDING**			
Funding	20	Describe sources of funding for the systematic review and other support (e.g., supply of data); role of funders for the systematic review.	16

From: Moher D, Liberati A, Tetzlaff J, Altman DG, The PRISMA Group (2009). Preferred Reporting Items for Systematic Reviews and Meta-Analyses: The PRISMA Statement. PLoS Med 6(7): e1000097. doi:10.1371/journal.pmed1000097

Supplementary Table 2. PubMed search strategy

1	severe acute respiratory syndrome coronavirus 2[Supplementary Concept]
2	severe acute respiratory syndrome coronavirus 2[Text Word]
3	SARS-CoV-2[Text Word]
4	sars cov 2[Text Word]
5	novel corona virus[Text Word]
6	new coronavirus[Text Word]
7	novel coronavirus[Text Word]
8	2019-nCoV[Text Word]
9	2019nCoV[Text Word]
10	2019 novel CoV[Text Word]
11	2019 nCoV[Text Word]
12	2019 novel coronavirus[Text Word]
13	2019 novel coronavirus disease[Text Word]
14	"Wuhan"[Text Word] AND ("coronavirus"[MeSH Terms] or "coronavirus"[Text Word])
15	novel coronavirus pneumonia[Text Word]
16	NCP[Text Word]
17	corona virus disease 2019[Text Word]
18	coronavirus disease 19[Text Word]
19	coronavirus disease 2019[Text Word]
20	coronavirus disease-19[Text Word]
21	COVID-19[Supplementary Concept]
22	COVID-19[Text Word]
23	COVID 19[Text Word]

24	COVID19[Text Word]
25	COVID-2019[Text Word]
26	Novel Coronavirus-Infected Pneumonia[Text Word]
27	NCIP[Text Word]
28	or/1-27
29	"2019/12/01"[Date - Entrez] : "3000"[Date - Entrez]
30	20 and 30
31	"animals"[MeSH Terms] NOT "humans"[MeSH Terms]
32	30 not 31

Supplementary Table 3. Characteristics of included studies in the meta-analysis

First author	Centre	Period	Location	Participants	Normal range of AST (U/L)	Normal range of ALT (U/L)	Subgroup	Sample size	Age (mean ± SD or Median (IQR or Range))	Sex (Male, %)	Pre-existing liver diseases (n, %)
Arentz, M. [14]	Single	2020/2/20-2020/3/5	Washington State, USA	Critically ill Patients	5-40	5-50	NA	21	70 (Range:43-92)	11(52)	Cirrhosis:1 (4.8)；Abnormal Liver function:8 (38).
Bhatraju, P. K.[27]	Multi	2020/2/24-2020/3/9	Seattle Region, USA	Critically ill Patients	≤40	≤40	NA	24	64±18 (Range:23-97)	15(63)	NR
Cai, J.[28]	Multi	2020/1/19-2020/2/3	Shanghai, Hainan, Hefei, and Qingdao, China	Children	15-40	9-50	NA	10	74 months (Range:3-131 months)	4(40)	NR
Cai, Q.[29]	Single	2020/1/11-2020/2/6	Shenzhen, China	COVID-19	0-40	0-40	Total	298	47.5 (33-61)	145 (48.66)	28 (9.4)
							Non-severe	240	41 (31-56)	106 (44.17)	20 (8.33)

							Severe	58	62.5(56-66)	39 (67.24)	8 (13.79)
Cao, B.[30]	Single	2020/1/18-2020/2/3	Wuhan, China	Adults	≤40	≤50	Total	199	58 (49–68)	125 (60.3)	Exclusion criteria include severe liver disease.
							Lopinavir–ritonavir	99	58 (50–68)	61 (61.6)	
							Standard care	100	58 (48–68)	59 (59.0)	
Cao, J.[31]	Single	2020/1/3-2020/2/1	Wuhan, China	Adults	NR	＜40	Total	102	54 (37-67)	53 (52)	2 (2)
							Non-survivor	17	72 (63-81)	13 (76.5)	1 (5.9)
							Survivor	85	53 (47-66)	40 (47.1)	2 (2.4)
Chen, G.[79]	Single	2019/12-2020/1/27	Wuhan, China	COVID-19	≤40	≤41	Total	21	56 (50-65)	17 (81)	NR
							Severe	11	61 (56.5-66)	10 (90.9)	NR
							Moderate	10	52 (42.8-56)	7 (70)	NR
Chen, L.[32]	Single	2020/1/14-2020/1/29	Wuhan, China	Adults	≤40	≤41	NA	29	56 (Range:26-79)	21 (72)	2 (6)
Chen, N.[4]	Single	2020/1/1-2020/1/20	Wuhan, China	Adults	15-40	9-50	NA	99	55.5±13.1	67 (67.67)	NR
Chen, T.[78]	Single	2020/1/13-2020/2/28	Wuhan, China	COVID-19	≤40	≤41	Total	274	62 (44-70)	171 (62)	11 (4)

							Deaths	113	68 (62-77)	83 (73)	5 (4)
							recovered	161	51 (37-66)	88 (55)	6 (4)
Chen, T.[33]	Single	2020/1/1-2020/2/20	Wuhan, China	COVID-19	15-40	9-50	Total	203	54 (20-91)	108 (53.2)	8 (3.9)
							＜65y	148	46 (20-64)	74 (50)	6 (4.1)
							⩾65y	55	74 (65-91)	34 (61.8)	2 (3.6)
Chen, X.[34]	Single	2020/1/26-2020/1/31	Chongqing, China	COVID-19	⩽35	⩽40	NA	78	45 (Range 15-79)	39 (50)	NR
Du, R. H.[35]	Multi	2019/12/25-2020/2/15	Wuhan, China	Decedents	⩽40	⩽50	Total	109	70.7±10.9	74 (67.9)	2 (1.8)
							ICU	51	68.4±9.7	36(70.6)	0
							Non-ICU	58	72.7±11.6	38(65.5)	2(3.4)
Du, Y.[36]	Multi	2020/1/9-2020/2/15	Wuhan, China	Decedents	17-59	21-72	NA	85	65.8±14.2	62 (72.9)	5 (5.9)
Guan, W. J.[37]	Multi	2019/12/11-2020/1/31	Mainland, China	COVID-19	⩽40	⩽40	Total	1099	47 (35–58)	637/1096 (58.1)	NR
							Non-severe	926	45 (34–57)	537/923 (58.2)	NR
							Severe	173	52 (40–65)	73/173	NR

										(57.8)	
Guo, T.[38]	Single	2020/1/23-2020/2/23	Wuhan, China	COVID-19	NR	NR	Total	187	58.5±14.66	91 (48.7)	NR
							Normal TnT level	135	53.53±13.22	57 (42.2)	NR
							Elevated TnT level	52	71.4±9.43	34 (65.4)	NR
Han, X.[39]	Single	2019/12/20-2020/2/2	Wuhan, China	Adults	NR	NR	NA	17	40±10(Range:27-60)	6 (35.3)	NR
Han, Y. N.[40]	Single	2020/1/31-2020/2/16	Xi'an, China	COVID-19	Child: 10 - 50 Adult: 15 - 40	Child: 0 - 37 Adult: 9 - 50	Total	32	NR	16 (50)	NR
							Children	7	1.3 (Range 0.2 - 13)	4 (57.1)	NR
							Adults	25	44 (Range:22 - 70)	12 (48)	NR
He, X. W.[41]	Single	2020/2/3-2020/2/24	Wuhan, China	severe or critically ill	＜40	＜41	Total	54	68 (59.3-74.3)	34 (63)	NR

				patients							
							Death	26	70 (62.5-76.5)	16 (61.5)	NR
							Survivor	28	66.5 (56-71.8)	18 (64.3)	NR
Hu, Z.[42]	Multi	2020/1/28-2020/2/9	Nanjing, China	Patients with asymptomatic infection	≤40	≤40	Total	24	32.5 (19- 57)	8 (33.3)	0 (0)
							Cases with symptoms after diagnosis	5	53 (23- 65)	0 (0)	0 (0)
							Cases without symptoms after diagnosis	19	32 (15- 57)	8 (42.1)	0 (0)
							Asymptomatic plus CT normal cases	7	14 (6- 32)	3 (42.9)	0 (0)
							Other cases	17	38 (27- 65)	5 (29.4)	0 (0)

Huang, C. [1]	Single	2019/12/16-2020/1/2	Wuhan, China	COVID-19	≤40	NR	Total	41	49 (41-58)	30 (73)	1 (2)
							ICU care	13	49 (41-61)	11 (85)	0 (0)
							No ICU care	28	49 (41-57.5)	19 (68)	1 (4)
Huang, Y.[43]	Single	2019/12/21-2020/1/28	Wuhan, China	COVID-19	NR	NR	NA	34	56.24±17.14	14 (41.2)	1 (2.9)
Ji, D.[44]	Multi	2020/1/20-2020/2/17	Beijing, China	COVID-19	NR	Male: ≤30; Female: ≤19	Total	202	44.5 (34.8-54.1)	113 (55.9)	NR
							Stable	163	42.9 (32.6-51.8)	86 (52.8)	NR
							Progressive	39	55.1 (43.7-71.8)	27 (69.2)	NR
							GI	74	46.14±14.19	37 (50)	8 (10.81)
							non-GI	577	45.09±14.45	294 (50.95)	17 (2.95)
Khan, S.[12]	Single	2020/1/25-2020/2/15	Wuhan, China	Pregnant women	≤35	≤45	NA	17	29.29 (Range:24-34)	0 (0)	NR
Kim, E. S.[45]	Multi	2020/1/19-2020/2/17	Republic of Korea	Adults	NR	≤40	NA	28	42.6±13.4	15 (53.6)	1 (3.6)

Lei, Z.[46]	Multi	2020/1/22-2020/2/12	Guangzhou and Wuhan, China	Adults	NR	NR	Total	119	NR	NR	NR
							Guangzhou	20	43.2±14.0	10 (50)	NR
							Wuhan	99	55.5±13.1	67 (67.7)	NR
Li, Y. K.[47]	Single	2020/1/1-2020/2/20	Wuhan, China	Adults	NR	NR	Total	25	NR	NR	NR
							Hospitalized patients	13	60.2±5.6	10 (76.9)	NR
							Health care staff	12	35.8±9.2	2 (16.7)	NR
Lian, J.[48]	Multi	2020/1/17-2020/2/12	Zhejiang, China	COVID-19	15-40	9-50	Total	788	NR	NR	NR
							< 60y	652	41.15±11.38	349 (53.5)	25 (3.83)
							⩾ 60y	136	68.28±7.314	58 (42.6)	6 (4.41)
Lin, L.[49]	Single	2020/1/17-2020/2/15	Zhuhai, China	COVID-19	Male:15-40; Female: 13-35.	Male:9-50; Female: 7-40.	Total	95	45.3±18.3	45 (47.4)	NR

							With GI symptoms	58	48.0±17.1	27 (46.6)	NR
							Without GI symptoms	37	41.1±19.5	18 (48.6)	NR
Liu, C.[50]	Multi	2020/1/23-2020/2/8	Lanzhou, Shenyang, Ankang, Lishui, Zhenjiang, Baoding, Linxia, China	COVID-19	≤40	≤40	NA	32	38.5 (26.25-45.75)	20（62.5)	NR
Liu, M.[51]	Single	2020/1/10-2020/1/31	Wuhan, China	Medical staff	≤40	≤50	NA	30	35±8(Range:21-59)	10 (33.3)	1 (3.13)
Lo, I. L.[52]	Single	2020/1/21-2020/2/16	Macau, China	COVID-19	≤41	≤40	NA	10	54 (27-64)	3 (30)	NR
Lu X.[53]	Single	2020/1/28-2020/2/26	Wuhan, China	Children	10-50	7-45	Total	171	6.7 (2.0-9.8)	104 (60.8)	NR
							Asymptomatic infection	27	9.6 (7.6-12.6)	NR	NR
							Upper respiratory tract infection	33	3.9 (1.4-8.4)	NR	NR

							Pneumonia	111	5.9 (1.2-9.3)	NR	NR
Mi, B.[54]	Multi	2020/1/1-2020/2/27	Wuhan, China	Adults (Fracture Patients)	8-40	5-35	NA	10	76 (Range:34-87)	2 (20)	1 (10)
Qian, G. Q.[55]	Multi	2020/1/20-2020/2/11	Zhejiang, China	COVID-19	15-40	9-50	NA	91	50 (36.5-57)	37 (40.66)	NR
Qian, Z. P.[56]	Single	2020/1/20-2020/2/24	Shagnhai, China	COVID-19	≤35	≤40	Total	324	51 (36-64)	167 (51.5)	70 (21.6)
							Mild	298	48.5 (35-63)	147 (49.3)	61 (20.5)
							Severe	26	65 (63-76)	20 (76.9)	9 (34.6)
Qiu, H.[6]	Multi	2020/1/17-2020/3/1	Zhejiang, China	Children	<40	<40	Total	36	8.3±3.5	23 (64)	NR
							Mild cases	17	7.5±3.2	10 (59)	NR
							Moderate cases	19	9±3.6	13 (68)	NR
Qiu, L.[57]	Single	2020/2/4-2020/2/24	Wuhan, China	Adult women, ICU	14-36	9-52	NA	10	66 (Range: 52-80)	0 (0)	NR
Shi, H.[58]	Multi	2019/12/20-2020/1/23	Wuhan, China	COVID-19	≤40	NR	NA	81	49.5±11	42 (52)	7 (9)

To, K. K.[5]	Multi	2020/1/22-2020/2/12	Hong Kong, China	COVID-19	NR	≤53	Total	23	62(Range:37-75)	NR	NR
							Severe	10	66 (Range:39-75)	6 (60)	NR
							Mild	13	56 (Range:37-75)	7 (54)	NR
Tu, W. J.[59]	Single	2020/1/3-2020/2/24	Wuhan, China	COVID-19	NR	NR	Total	174	NR	NR	NR
							Non-survivors	25	70 (64-80)	19 (76)	NR
							Discharge	149	51 (37-62)	60 (40)	NR
Wan, S.[60]	Single	2020/1/23-2020/2/8	Chongqing, China	COVID-19	≤40	NR	Total	135	47 (36 - 55)	72 (53.3)	2 (1.5)
						NR	Mild	95	44 (33 - 49)	52 (54.7)	1 (1)
						NR	Severe	40	56 (52 - 73)	21 (52.5)	1 (1)
Wang, D.[61]	Multi	2020/1/25-2020/2/21	Shanxi, Gansu, Ningxia, Hebei, Henan, Shandong, China	Children	NR	NR	NA	31	7 y 1 m (Range:6m-17y)	15 (48.4)	NR
Wang, F.[62]	Single	2020/1/20-2020/2/28	Wuhan, China	COVID-19	15-40	9-50	Total	52	NR	NR	NR
							with pancreatic injury	9	55 ± 15	6 (67)	NR

							without pancreatic injury	43	52 ± 18	18 (42)	NR
Wang, L.[63]	Single	2020/1/21-2020/2/5	Zhengzhou, China	COVID-19	NR	NR	NA	18	39 (29 - 55)	10 (55.6)	NR
Wang, Y.[15]	Single	2020/1/25-2020/2/25	Wuhan, China	critically ill	⩽41	⩽41	Total	344	64 (52-72)	179 (52)	NR
							Survivors	211	57 (47-69)	105 (49.8)	NR
							Non-survivors	133	70 (62-77)	74 (55.6)	NR
Wang, Z.[64]	Single	2020/1/16-2020/1/29	Wuhan, China	COVID-19	⩽40	⩽35	Total	69	42 (35–62)	32 (46)	1 (1)
							SpO2 ⩾ 90%	55	37 (32–51)	25 (45)	1 (2)
							SpO2 < 90%	14	70.5 (62–77)	7 (50)	0 (0)
Wu, J.[65]	Multi	2020/1/22-2020/2/14	Jiangsu, China	COVID-19	15-40	9-50	NA	80	46.10±15.42	39 (48.75)	1 (1.25)
Xu, X. W.[66]	Multi	2020/1/10-2020/1/26	Zhejiang, China	COVID-19	13-35	7-40	Total	62	41 (32-52)	35 (56)	7 (11)
							Time since symptom onset>10 days	33	45 (37-54)	19 (58)	4 (12)
							Time since	29	39 (31-50)	16 (55)	3 (10)

							symptom onset ≤10 days				
Xu, Y.[67]	Single	2020/1/22-2020/2/20	Guangzhou, China	Children	5-60	9-50	NA	10	7.54±5.92	6 (60)	NR
Yang, W.[68]	Multi	2020/1/17-2020/2/10	Wenzhou, China	COVID-19	8-40	0-64	NA	149	45.11±13.35	81 (54.36)	NR
Yao, N.[70]	Single	2020/1/21-2020/2/21	Shanxi, China	COVID-19	≤46	≤66	Total	40	53.87±15.84	25 (62.5)	NR
							Liver injury	22	NR	14 (63.6)	NR
							Non- liver injury	18	NR	10 (57.1)	NR
Yang, X.[69]	Single	2019/12/24-2020/1/26	Wuhan, China	critically ill	NR	NR	Total	52	59.7±13.3	35(67)	Liver dysfunction 15 (29)
							Survivors	20	51.9±12.9	14(70)	Liver dysfunction 6 (30)
							Non-survivors	32	64.6±11.2	21(66)	Liver dysfunction 9 (28)
Zha, L.[71]	Multi	2020/1/24-2020/2/24	Wuhu, China	COVID-19	NR	NR	Total	31	39 (32-54)	20 (64)	2 (6)
							Non-corticosteroid	20	37 (27-52)	12 (60)	2 (10)
							Corticosteroid	11	53 (36-57)	8 (73)	0 (0)

Zhang, G.[72]	Single	2020/1/16-2020/2/25	Wuhan, China	COVID-19	< 40	< 40	Total	95	49 (39-58)	53 (55.8)	NR
							No-severe	63	49 (41-57)	32 (50.8)	NR
							Severe	32	50.5 (38.3-58.8)	21 (65.6)	NR
							Normal imaging	72	34.9±14.2	33 (45.8)	2 (2.8)
							Abnormal imaging	573	46.65±13.82	295 (51.5)	23 (4)
Zhang, Y.[73]	Single	2020/1/18-2020/2/22	Wuhan, China	COVID-19	≤40	≤50	Total	115	49.52±17.06	57 (49)	NR
							Mild	84	43.96±14.84	34 (29)	NR
							Severe	31	64.58±13.26	23 (20)	NR
Zhao, D.[74]	Multi	2020/1/23-2020/2/5	Anhui, China	COVID-19	15-40	9-50	NA	19	48 (27-56)	11 (57.89)	1 (5.26)
Zheng, F.[75]	Single	2020/1/23-2020/2/5	Changsha, China	COVID-19	≤40	≤40	Total	161	45 (33.5-57)	80 (49.7)	4 (2.5)
							Non-severe	131	40 (31-51)	66 (50.4)	4 (3.1)
							Severe	30	57 (46.5-66)	14 (46.7)	0 (0)
Zhou, F.[76]	Multi	2019/12/29-2020/1/31	Wuhan, China	Adults	NR	≤40	Total	191	56 (46–67)	119 (62)	NR

							Non-survivor	54	69 (63–76)	38 (70)	NR
							Survivor	137	52 (45–58)	81 (59)	NR
Zhu, L.[77]	Multi	2020/1/24-2020/2/22	Jiangsu, China	children	NR	⩽40	NA	10	9.15±4.67	5 (50)	NR

Abbreviations: NA, not applicable; NR, not reported; ICU, intensive care unit; CT, computed tomography; GI, gastrointestinal; SpO_2, oxygen saturation.

Note: Age was presented as mean ± SD or median (IQR or Range), "Range" is intentionally marked.

Supplementary Table 4. Quality evaluation of all the included studies.

Study	Was the sample representative of the target population?	Were study participants recruited in an appropriate way?	Was the sample size adequate?	Were the study subjects and the setting described in detail?	Was data analysis conducted with sufficient coverage of the identified sample?	Were valid methods used for the identification of the condition?	Was the condition measured in a standard, reliable way for all participants?	Was there appropriate statistical analysis?	Was the response rate adequate, and if not, was the low response rate managed appropriately?
Arentz, M. [14]	Yes	Unclear	No	Yes	Yes	Yes	Yes	Yes	Yes
Bhatraju, P. K. [27]	Yes	Unclear	No	Yes	Yes	Yes	Yes	Yes	Yes
Cai, J. [28]	No	Unclear	No	Yes	Yes	Yes	Yes	Not applicable	Yes
Cai, Q. [29]	Yes	Unclear	Yes	Yes	Yes	Yes	Yes	Yes	Yes
Cao, B. [30]	Yes	Unclear	No	Yes	Yes	Yes	Yes	Yes	Yes
Cao, J. [31]	Yes	Unclear	No	Yes	Yes	Yes	Yes	Yes	Yes
Chen, G. [79]	Yes	Unclear	No	Yes	Yes	Yes	Yes	Yes	Yes
Chen, L. [32]	Yes	Unclear	No	Yes	Yes	Yes	Yes	Yes	Yes
Chen, N. [4]	Yes	Unclear	No	Yes	Yes	Yes	Yes	Yes	Yes
Chen, T. [78]	Yes	Unclear	No	Yes	Yes	Yes	Yes	Yes	Yes

Chen, T. [33]	Yes	Unclear	No	Yes	Yes	Yes	Yes	Yes	Yes
Chen, X. [34]	No	Unclear	No	Yes	Yes	Yes	Yes	Yes	Yes
Du, R. H. [35]	Yes	Unclear	No	Yes	Yes	Yes	Yes	Yes	Yes
Du, Y. [36]	Yes	Yes	No	Yes	Yes	Yes	Yes	Yes	Yes
Guan, W. J. [37]	Yes	No	Yes	Yes	No	Yes	Yes	Yes	No
Guo, T. [38]	Yes	Yes	No	Yes	Yes	Yes	Yes	Yes	Yes
Han, X. [39]	Yes	Unclear	No	Yes	Yes	Yes	Yes	Yes	Yes
Han, Y. N. [40]	Yes	Unclear	No	Yes	Yes	Yes	Yes	Yes	Yes
He, X. W. [41]	Yes	Unclear	No	Yes	Yes	Yes	Yes	Yes	Yes
Hu, Z. [42]	Yes	Unclear	No	Yes	Yes	Yes	Yes	Yes	Yes
Huang, C. [1]	Yes	Unclear	No	Yes	Yes	Yes	Yes	Yes	Yes
Huang, Y. [43]	Yes	Unclear	No	Yes	Yes	Yes	Yes	Yes	Yes
Ji, D. [44]	Yes	Yes	No	Yes	Yes	Yes	Yes	Yes	Yes
Khan, S. [12]	No	Unclear	No	Yes	Yes	Yes	Yes	Yes	Yes

Kim, E. S.[45]	Yes	Unclear	No	Yes	Yes	Yes	Yes	Yes	Yes
Lei, Z. [46]	Yes	Unclear	No	Yes	Yes	Yes	Yes	Yes	Yes
Li, Y. K. [47]	Yes	Unclear	No	Yes	Yes	Yes	Yes	Yes	Yes
Lian, J. [48]	Yes	Unclear	Yes	Yes	Yes	Yes	Yes	Yes	Yes
Lin, L. [49]	Yes	Unclear	No	Yes	Yes	Yes	Yes	Yes	Yes
Liu, C. [50]	Yes	Yes	No	Yes	Yes	Yes	Yes	Yes	Yes
Liu, M. [51]	No	Unclear	No	Unclear	Yes	Yes	Yes	Yes	Yes
Lo, I. L.[52]	Yes	Unclear	No	Yes	Yes	Yes	Yes	Yes	Yes
Lu X. [53]	No	Unclear	No	Yes	Yes	Yes	Yes	Yes	Yes
Mi, B. [54]	No	Unclear	No	Yes	Yes	No	Yes	Yes	Yes
Qian, G. Q [55]	Yes	Unclear	No	Yes	Yes	Yes	Yes	Yes	Yes
Qian, Z. P [56]	Yes	Unclear	Yes	Yes	Yes	Yes	Yes	Yes	Yes
Qiu, H [6]	No	Unclear	No	Yes	Yes	Yes	Yes	Yes	Yes
Qiu, L.[57]	No	Unclear	No	Yes	Yes	Yes	Yes	Not applicable	Yes

Shi, H. [58]	Yes	Unclear	No	Yes	Yes	Yes	Yes	No	Yes
To, K. K. [5]	Yes	Yes	No	Yes	Yes	Yes	Yes	Yes	Yes
Tu, W. J. [59]	Yes	Yes	No	Yes	Yes	Yes	Yes	Yes	Yes
Wan, S. [60]	Yes	Unclear	No	Yes	Yes	Yes	Yes	Yes	Yes
Wang, D. [61]	No	Unclear	No	Yes	Yes	Yes	Yes	Not applicable	Yes
Wang, F. [62]	Yes	Unclear	No	Yes	Yes	Yes	Yes	Yes	Yes
Wang, L. [63]	Yes	Unclear	No	Yes	Yes	Yes	Yes	Yes	Yes
Wang, Y. [15]	Yes	Unclear	Yes	Yes	Yes	Yes	Yes	Yes	Yes
Wang, Z. [64]	Yes	Yes	No	Yes	Yes	Yes	Yes	Yes	Yes
Wu, J. [65]	Yes	Unclear	No	Yes	Yes	Yes	Yes	Yes	Yes
Xu, X. W. [66]	Yes	Unclear	No	Yes	Yes	Yes	Yes	Yes	Yes
Xu, Y. [67]	No	Unclear	No	Yes	Yes	Yes	Yes	Not applicable	Yes
Yang, W. [68]	Yes	Yes	No	Yes	Yes	Yes	Yes	Yes	Yes
Yao, N. [70]	Yes	Unclear	No	Yes	Yes	Yes	Yes	Yes	Yes

Yang, X. [68]	Yes	Unclear	No	Yes	Yes	Yes	Yes	Yes	Yes
Zha, L. [71]	Yes	Unclear	No	Yes	Yes	Yes	Yes	Yes	Yes
Zhang, G. [72]	Yes	Unclear	No	Yes	Yes	Yes	Yes	No	Yes
Zhang, Y. [73]	Yes	Unclear	No	Yes	Yes	Yes	Yes	Yes	Yes
Zhao, D. [74]	Yes	Unclear	No	Yes	Yes	Yes	Yes	Yes	Yes
Zheng, F. [75]	Yes	Unclear	No	Yes	Yes	Yes	Yes	Yes	Yes
Zhou, F. [76]	Yes	Unclear	No	Yes	Yes	Yes	Yes	Yes	Yes
Zhu, L. [77]	No	Unclear	No	Yes	Yes	Yes	Yes	Not applicable	Yes

Note: The Joanna Briggs Institute (JBI) prevalence critical appraisal tool was used to perform quality evaluation. **Yes**, Meet the requirements of the quality evaluation; **No**, Not meet the requirements of the quality evaluation; **Unclear**, Unclear whether it meets the requirements or not; **Not applicable**, Not applicable to this quality evaluation.

According to the appraisal of the sample size, PASS 11.0 software was used to calculate the sample size required for the cross-sectional study on the COVID-19 related transaminase elevation. The prevalence of transaminase elevation in the COVID-19 patients was considered as 20% on the base of previous studies reported. The related confidence interval width (Two-Sided) was set as 10% with a power (1-β) estimate of 80%, and a type I error of 5% (α = 0.05, one-sided). Hence, a sample of 264 patients was required. Besides, the rate of the incomplete follow-up or dropout in the whole recruited patients was controlled below 10%. Ultimately, the sample should not be less than 293 in the cross-sectional study on the COVID-19 related transaminase elevation. If the sample size is below 293, it was not adequate.

Supplementary Table 5. Definition of liver injury in studies including liver injury

First author	Definition of liver injury
Arentz, M.[14]	Defined as an ALT or AST level greater than 3 times the ULN.
Cai, Q.[29]	Liver injury was defined by an ALT and/or AST higher than 3-fold of the ULN, or GGT and/or TBil higher than 2-fold of the ULN.
Chen, G.[79]	Acute liver injury was defined as jaundice with a TBil level of ⩾ 3 mg/dL and an acute increase in ALT of at least five times the upper limit of the normal range and/or an increase in ALP of at least twice the upper limit of the normal range.
Chen, T. [78]	Jaundice with a TBil level of ⩾ 3 mg/dl and an acute increase in ALT of at least five times the upper limit of the normal range and/or an increase in ALP of at least twice the upper limit of the normal range.
Ji, D.[44]	Liver injury was defined as hepatocellular if the ALT level was >30 IU/L for males and >19 IU/L for females; ductular if ALP was >ULN accompanied by GGT > ULN; mixed if both hepatocellular and ductular enzymes were raised >ULN.
Lin, L.[49]	The patients developed hepatic function impairment during hospitalisation with elevated bilirubin, aspartate transaminase or alanine aminotransferase.
Wang, F.[62]	Any abnormality in AST, ALT, GGT or ALP.
Wang, L.[63]	Patients had abnormal liver function, with ALT and AST above the normal range.
Wang, Y.[15]	Liver injury was diagnosed according to elevation of bilirubin and aminotransferase.
Yao, N.[70]	In the course of treatment, liver function examination with one or more of the following abnormalities is considered as liver injury: ALT > 66 U/L, AST > 46 U/L, TBil > 20.5 μmol/L.
Du, Y.[36]	NR
Guo, T.[38]	NR
Lian, J.[48]	The definition of liver damage was ALT >50 U/L or AST >40 U/L.

Tu, W. J.[59]	NR
Yang, X.[69]	NR
Zha, L.[71]	NR

Abbreviations: ALP, alkaline phosphatase; ALT, alanine aminotransferase; AST, aspartate aminotransferase; GGT, gamma-glutamyl transferase; NR, not reported; TBil, total bilirubin; ULN, upper limit of normal.

[a] Liver injury in the Lian study was defined according to the definition of the liver injury reported in the Jin article (Jin, X., et al. Gut, 2020, PMID: 32213556). The subjects in the Lian and Jin studies were from the same cohort of COVID-19 patients, and the corresponding authors of the two studies were the same. Hence, the Jin study was excluded.

Supplementary Figure 1 The prevalence of liver injury with strict definitions in severe patients

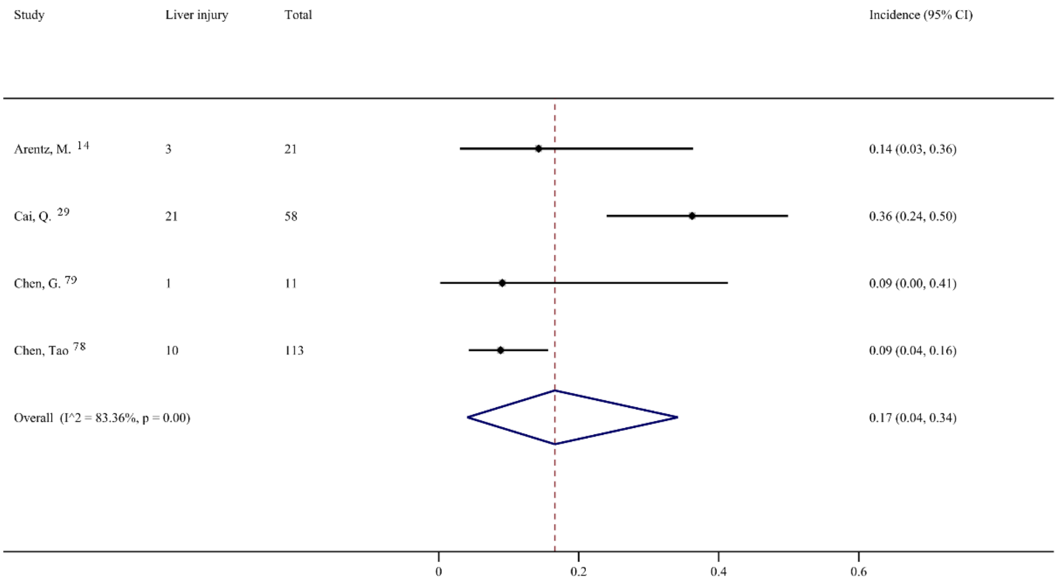

Supplementary Figure 2. Pooled estimate of elevated alanine aminotransferase in critically ill and fatal patients with COVID-19

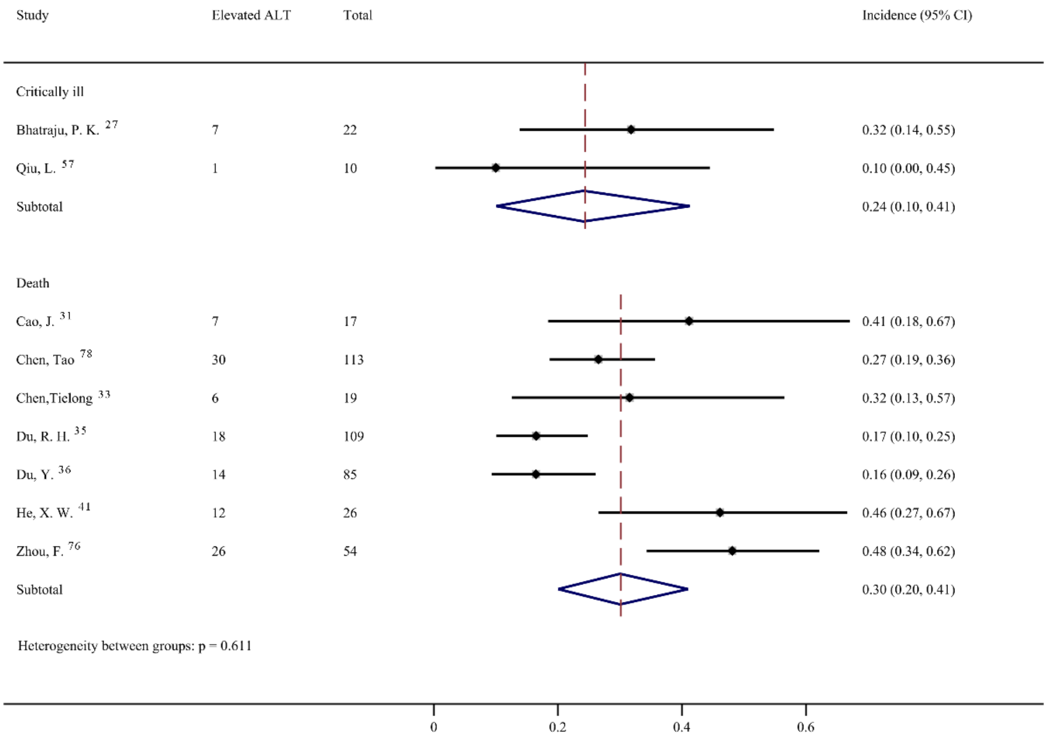

Supplementary Figure 3. Pooled estimate of elevated aspartate aminotransferase in critically ill and fatal patients with COVID-19

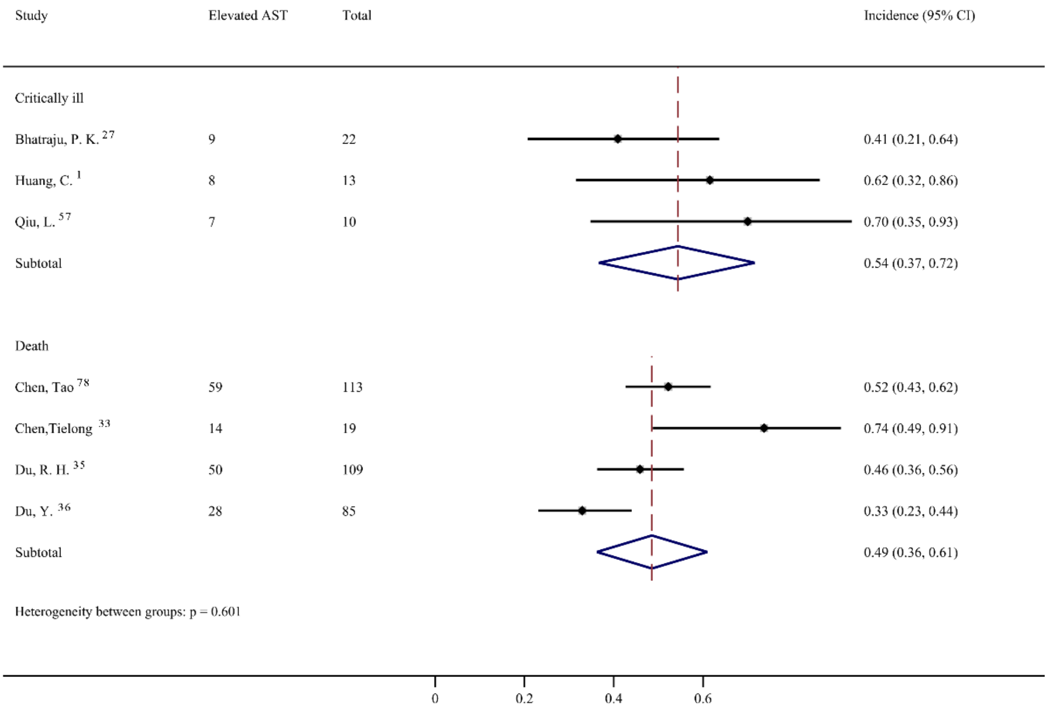

Supplementary Figure 4. Pooled estimate of elevated alanine aminotransferase in paediatric and adult patients with COVID-19

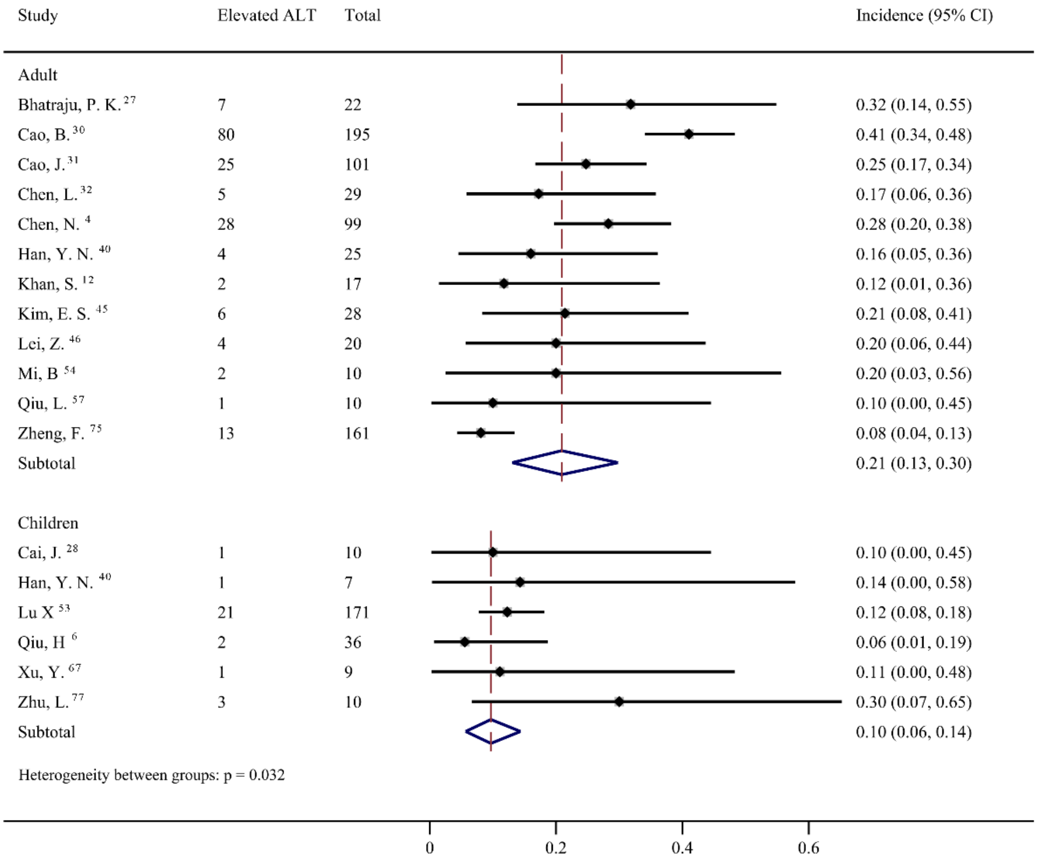

Supplementary Figure 5. Pooled estimate of elevated aspartate aminotransferase in paediatric and adult patients with COVID-19

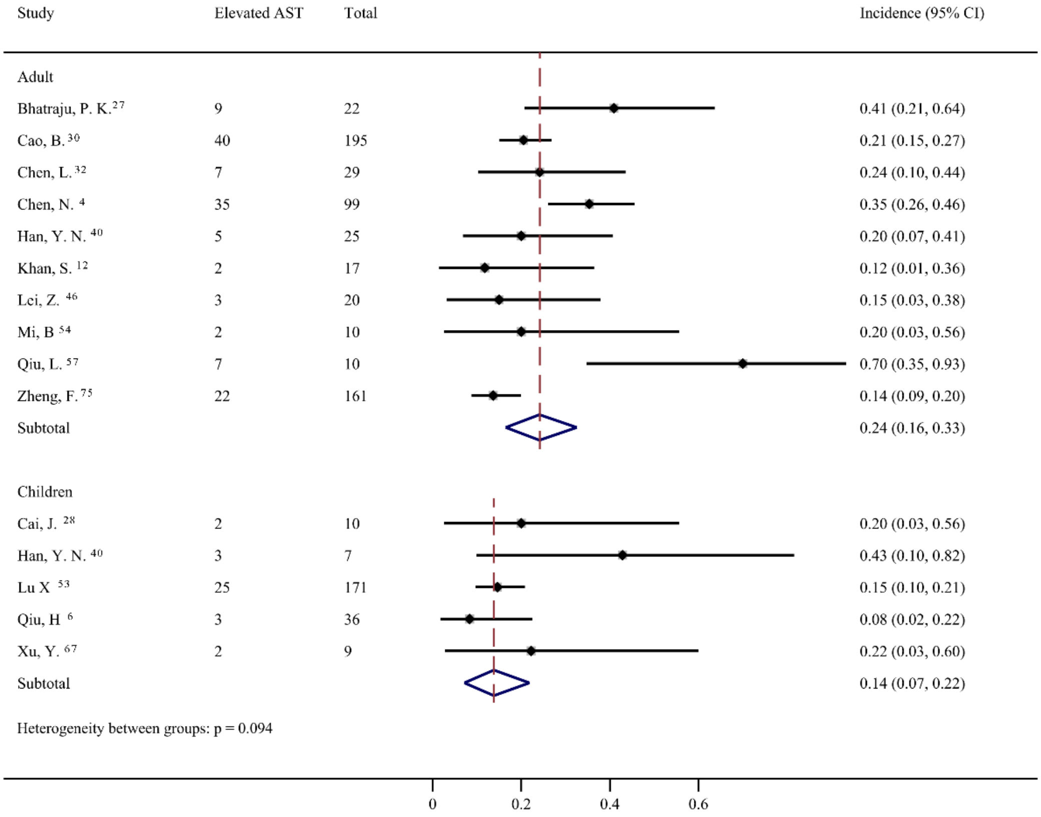

Supplementary Figure 6. Pooled estimate of elevated alanine aminotransferase in paediatric, severe and non-severe adult patients with COVID-19

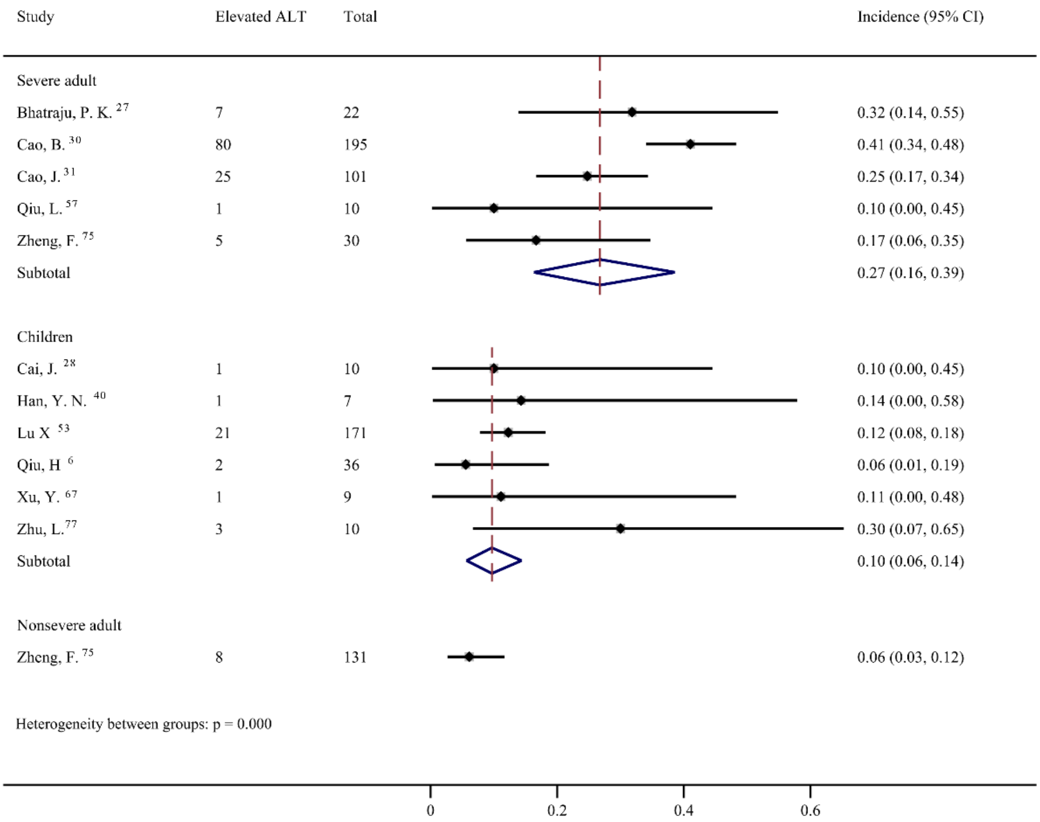

Supplementary Figure 7. Pooled estimate of elevated aspartate aminotransferase in paediatric, severe and non-severe adult patients with COVID-19

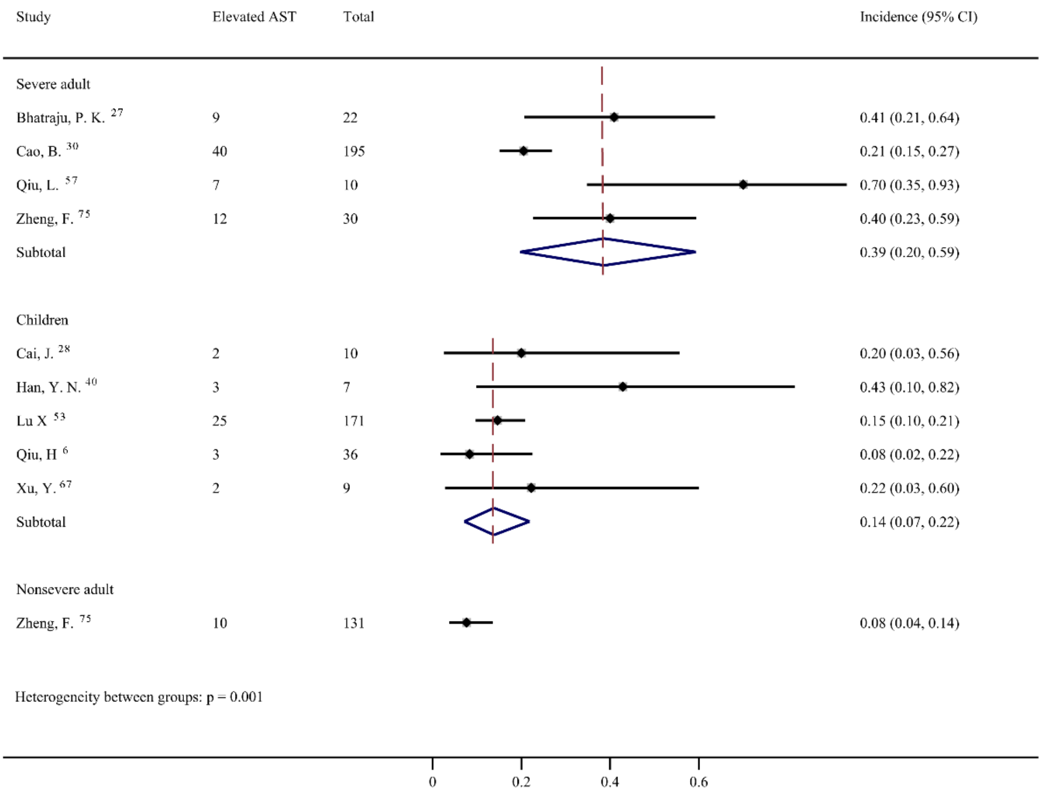

Supplementary Figure 8. The funnel plot of alanine aminotransferase

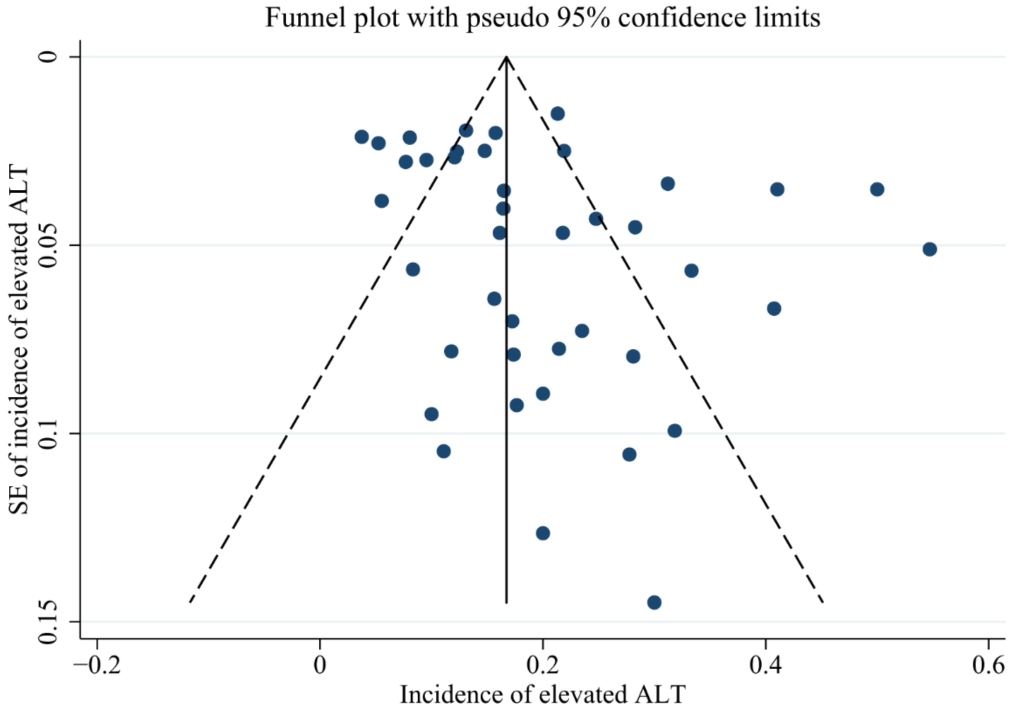

Supplementary Figure 9. The funnel plot of aspartate aminotransferase

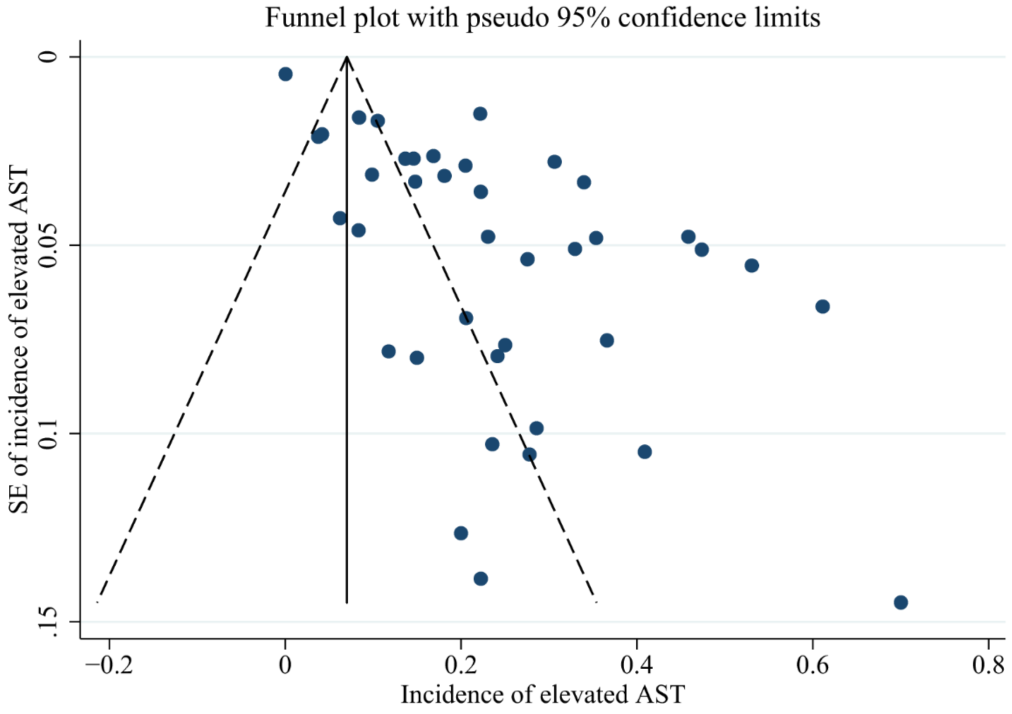

Supplementary Figure 10. The funnel plot of liver injury

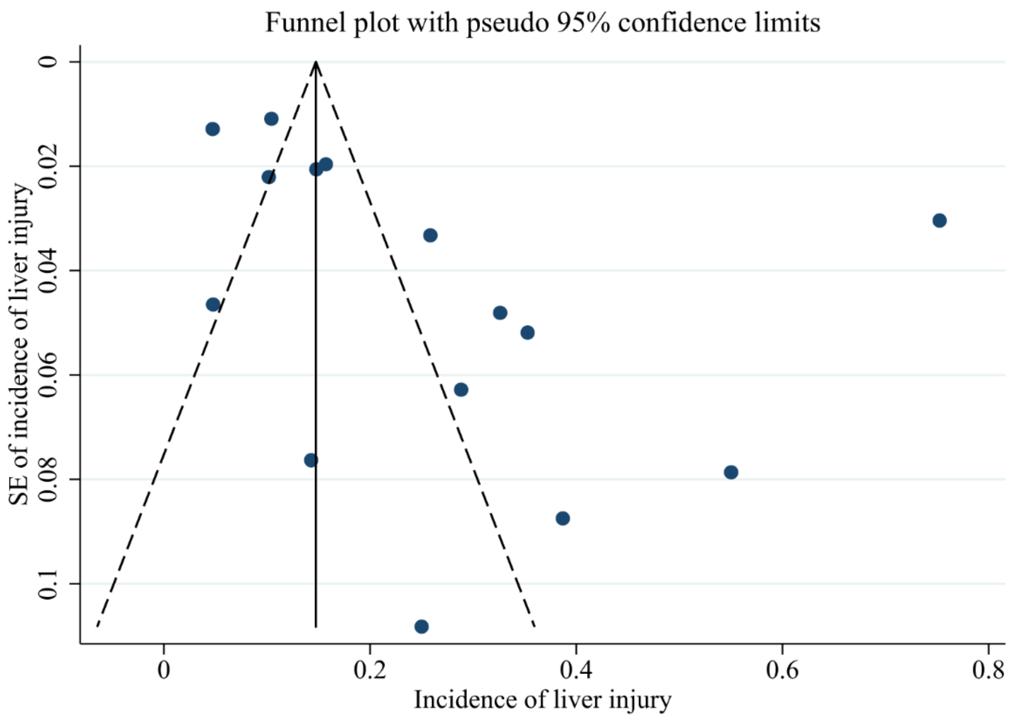

Supplementary Figure 11. The result of the alanine aminotransferase sensitivity analysis imputed by the trim and fill plot

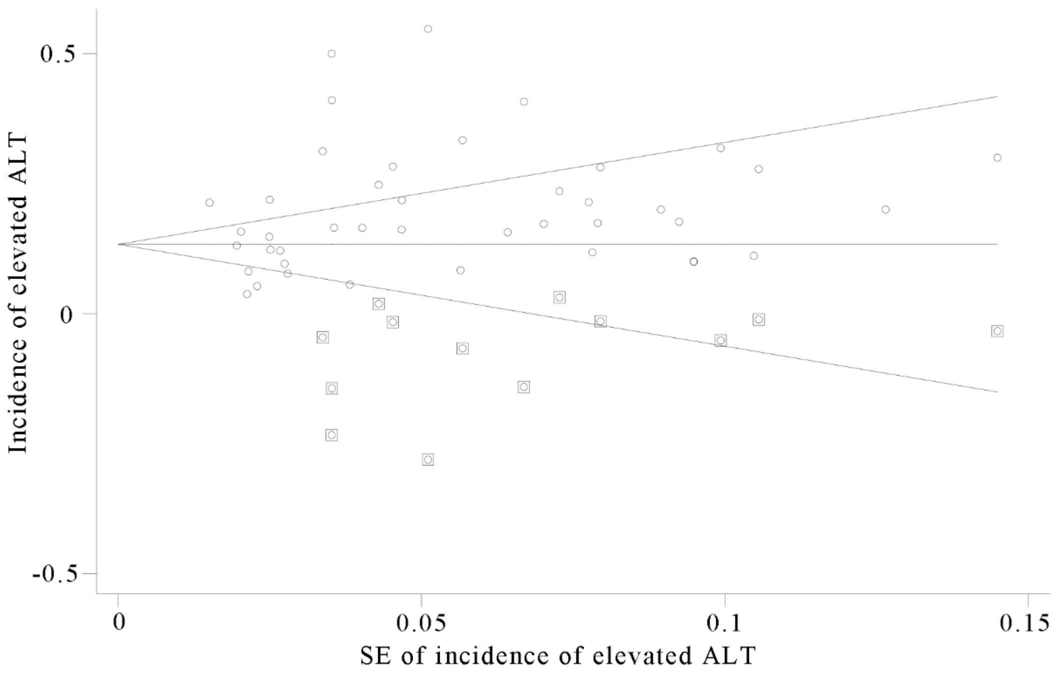

Supplementary Figure 12. The result of the aspartate aminotransferase sensitivity analysis imputed by the trim and fill plot

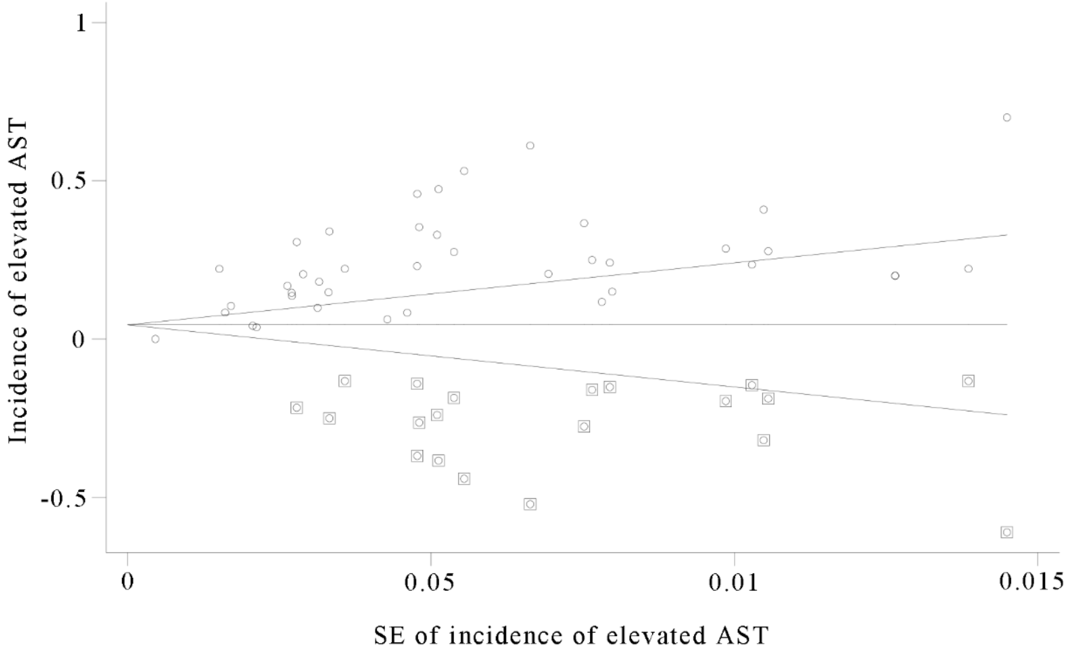

Supplementary Figure 13. The result of the liver injury sensitivity analysis imputed by the trim and fill plot

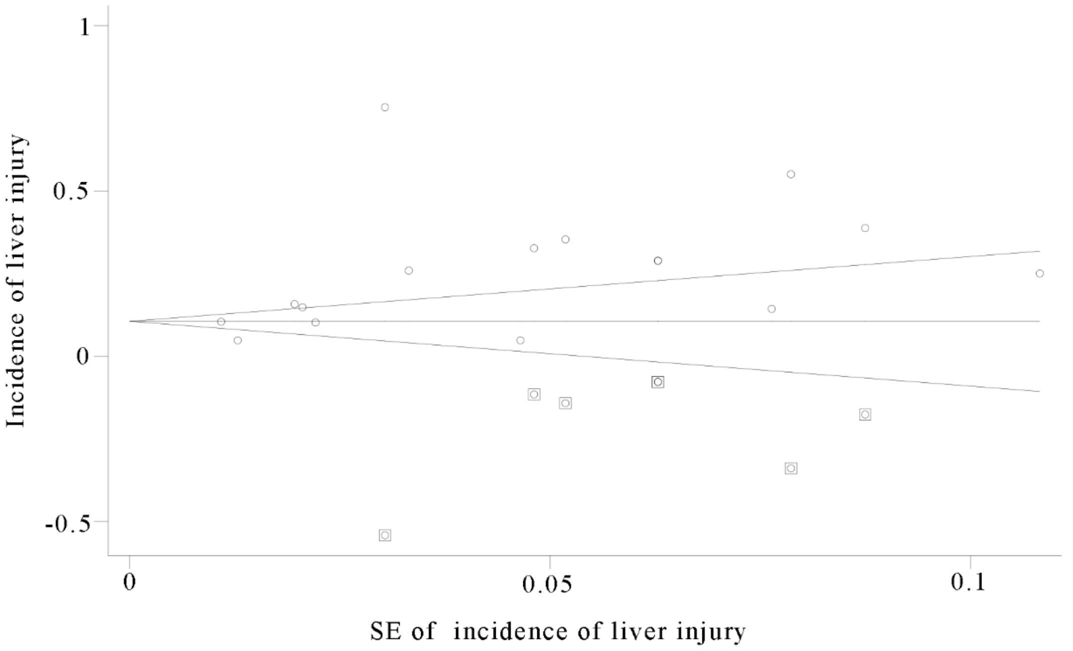

Supplement: Supplementary file 1 — Supplementary figures and tables. [file ijmsv18p1285s1.pdf]
